# Supplementary material for: Peptide translocation across asymmetric phospholipid membranes
Source: Biophys J. 2024 Feb 15;123(6):693–702. doi: 10.1016/j.bpj.2024.02.006 (PMC10995401; doi:10.1016/j.bpj.2024.02.006)
Supplement: Document S1. Figures S1–S22 and Tables S1–S5 [file mmc1.pdf]

Biophysical Journal, Volume *123*

Supplemental information

Peptide translocation across asymmetric phospholipid membranes

Ladislav Bartoš and Robert Vácha

SUPPORTING MATERIAL

Peptide translocation across asymmetric phospholipid membranes

Ladislav Bartoš and Robert Vácha

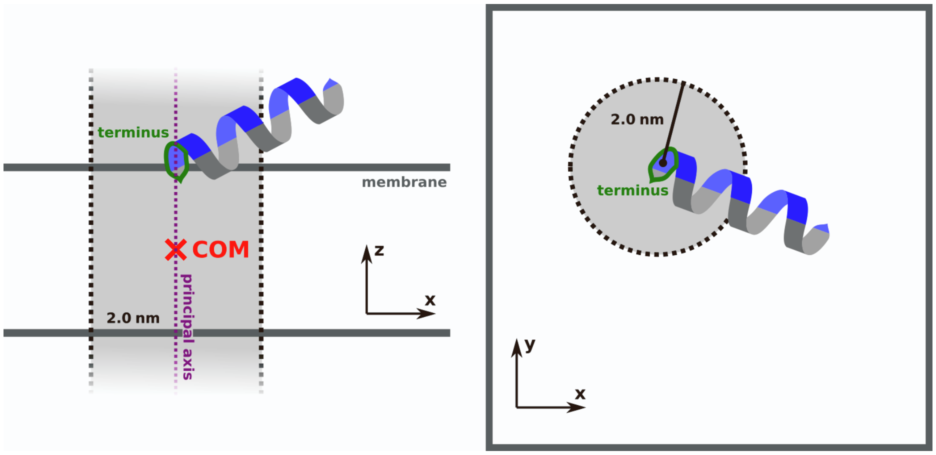

Figure S1: Schematic representation of the local membrane center of mass calculation. Left. Schematic side view of the membrane with peptide. Right. Schematic top view of the membrane with peptide. The local membrane center of mass (highlighted as red × symbol) is calculated from the positions of lipid beads localized inside a cylinder (highlighted in gray) with radius of 2.0 nm and its principal axis (purple dotted line) going along the z-axis through the center of mass of the inserting peptide terminus. Peptide terminus is defined as the first (N-terminus) or last (C-terminus) three backbone beads of the peptide and is highlighted in green. Note that the height of the cylinder (its dimension along the z-axis) is infinite.

Table S1: Distribution of umbrella sampling windows along the collective variable with biasing force constants used for insertion simulations of peptide translocation. Reference distances are in nm, force constants in kJ mol^{-1} nm^{-2}.

Reference distance	Force constant	Reference distance	Force constant
±2.50	1000*	±0.30	5000
±2.40	1000†	±0.25	5000
±2.30	1000#	±0.20	5000
±2.20	1000#	±0.15	5000
±2.10	1000#	±0.10	5000
±2.00	1000	±0.05	5000
±1.90	1000	0.00	5000
±1.80	1000	∓0.05	5000
±1.70	1000	∓0.10	5000
±1.60	1000	∓0.15	5000
±1.50	1000	∓0.20	5000
±1.45	3000	∓0.25	5000
±1.40	3000	∓0.30	5000
±1.35	3000	∓0.35	5000
±1.30	3000	∓0.40	5000
±1.25	3000	∓0.45	5000
±1.20	3000	∓0.50	3000
±1.15	3000	∓0.55	3000
±1.10	3000	∓0.60	3000
±1.05	3000	∓0.65	3000
±1.00	3000	∓0.70	3000
±0.95	3000	∓0.75	3000
±0.90	3000	∓0.80	3000
±0.85	3000	∓0.85	3000
±0.80	3000	∓0.90	3000
±0.75	3000	∓0.95	3000
±0.70	3000	∓1.00	1000
±0.65	3000	∓1.10	1000
±0.60	3000	∓1.20	1000
±0.55	3000	∓1.30	1000
±0.50	3000	∓1.40	1000
±0.45	5000	∓1.50	1000
±0.40	5000	∓1.60	1000*
±0.35	5000	∓1.70	1000*

* only used for symmetric DGPC, † only used for symmetric DGPC and asymmetric POPC/DGPC, # *not* used for symmetric PUPC

Table S2: Distribution of umbrella sampling windows along the collective variable with biasing force constants used for the adsorption simulations of peptide translocation. Reference distances are in nm, force constants in kJ mol^{-1} nm^{-2}.

Reference distance	Force constant	Reference distance	Force constant
±1.70	$1000^{\#}$	±3.50	500
±1.80	$1000^{\#}$	±3.60	500
±1.90	$1000^{\#}$	±3.70	500
±2.00	$1000^{*\dagger}$	±3.80	500
±2.10	1000^{*}	±3.90	500
±2.20	1000	±4.00	500
±2.30	1000	±4.10	500
±2.40	1000	±4.20	200
±2.50	1000	±4.40	200
±2.60	500	±4.60	200
±2.70	500	±4.80	200
±2.80	500	±5.00	200
±2.90	500	±5.20	200
±3.00	500	±5.40	200
±3.10	500	±5.60	200
±3.20	500	±5.80	200
±3.30	500	±6.00	200
±3.40	500		

$^{\#}$ only used for symmetric PUPC, * *not* used for symmetric DGPC, † *not* used for asymmetric POPC/DGPC

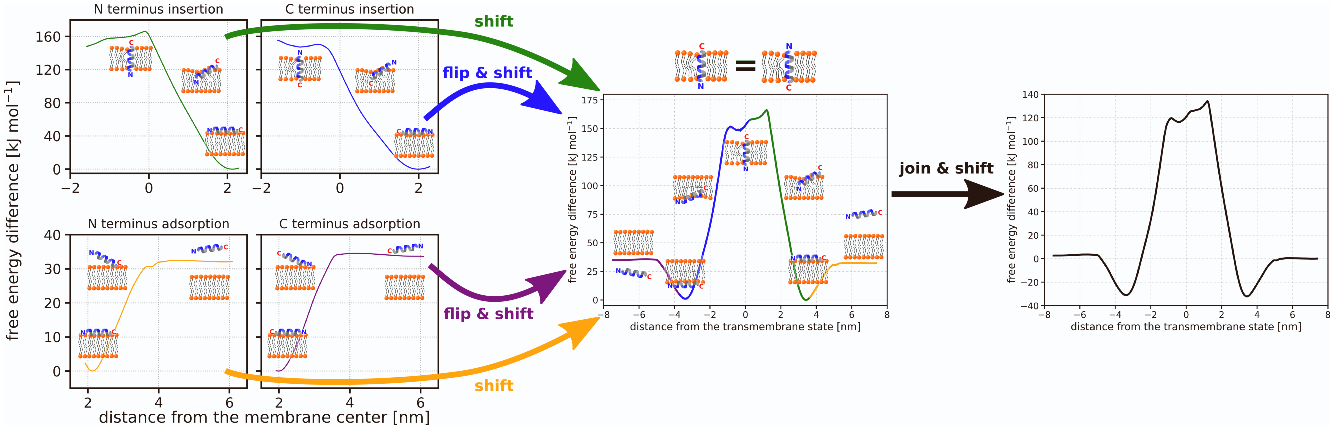

Figure S2: Example of the translocation profile construction demonstrated on free energy profiles calculated for the LK peptide translocating across symmetric POPE membrane. In symmetric membranes, the translocation process can be divided into four individual subprocesses: N-terminus insertion, C-terminus insertion, N-terminus adsorption, and C-terminus adsorption. To construct the complete translocation profile, we initially shift the profiles for N-terminus insertion and N-terminus adsorption in the x-dimension so that the transmembrane state of the peptide corresponds to $x = 0$. These aligned profiles are then joined with those for C-terminus insertion and adsorption. Since both N- and C-terminus insertion/adsorption are calculated using the peptide adsorbed on the same membrane leaflet, specifically the upper leaflet, the profiles for C-terminus insertion and adsorption must be flipped. This flipping is possible because the membrane is symmetric, and the leaflets are interchangeable. Thus, we flip the profiles for C-terminus insertion and adsorption, changing positive distance values to negative and vice versa. These flipped profiles represent insertion/adsorption from/to the lower leaflet. These profiles are again shifted in the x-dimension so that the transmembrane state is at $x = 0$. As illustrated in the middle chart, the free energy profiles of the individual subprocesses align almost perfectly, indicating only a small calculation error. We then combine the profiles and shift the ΔG values so that the free energy difference in the solvent (specifically in the solvent region of the N-terminus adsorption profile) equals zero. The free energy differences for peptide in solvent and in the adsorbed states on the opposite sides of the translocation profile provide a robust estimate of the error margin. It is important to note that in case of asymmetric membranes, the free energy profiles of subprocesses cannot be flipped. For asymmetric membranes, insertion/adsorption on both membrane leaflets is calculated, and appropriate free energy profiles are joined. The joining procedure for asymmetric membranes is otherwise equivalent to that described here.

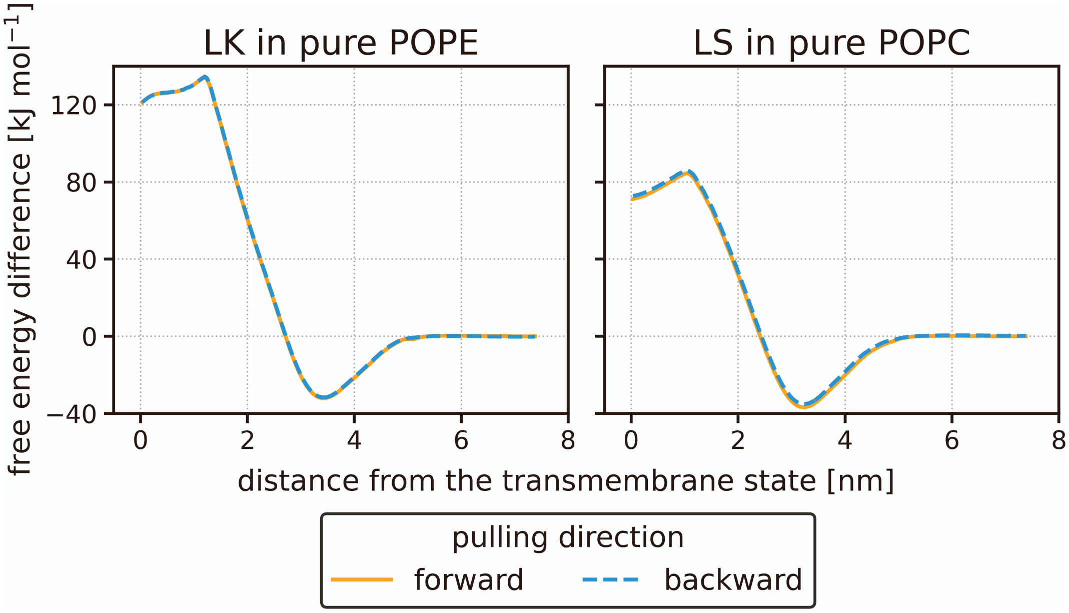

Figure S3: Free energy profiles of N-terminus insertion and adsorption of the LK peptide in a pure POPE membrane (left) and the LS peptide in a pure POPC membrane (right). The orange profiles were calculated from umbrella sampling windows which initial configurations were obtained from the standard "forward" pulling simulations. The blue dashed profiles were calculated from umbrella sampling windows with initial configurations from "backward" pulling simulations, performed in the opposite direction. For the LK and LS peptides, the maximum observed hysteresis was less than 1 kJ mol^{-1} and less than 2 kJ mol^{-1}, respectively. These values are significantly lower than our reported maximum error margin of 5 kJ mol^{-1}.

Table S3: Distribution of umbrella sampling windows along the collective variable with biasing force constants used for coarse-grained simulations of lipid flip-flop. Reference distances are in nm, force constants in kJ mol^{-1} nm^{-2}.

Reference distance	Force constant	Reference distance	Force constant
2.40	1000*	−0.05	4000
2.30	1000	−0.10	4000
2.20	1000	−0.15	3000
2.10	1000	−0.20	3000
2.00	1000	−0.25	3000
1.90	1000	−0.30	3000
1.80	1000	−0.35	2000
1.70	1000	−0.40	2000
1.60	1000	−0.45	2000
1.50	1000	−0.50	2000
1.40	1000	−0.55	2000
1.30	1000	−0.60	2000
1.20	1000	−0.65	2000
1.10	1000	−0.70	2000
1.00	2000	−0.75	2000
0.95	2000	−0.80	2000
0.90	2000	−0.85	2000
0.85	2000	−0.90	2000
0.80	2000	−0.95	2000
0.75	2000	−1.00	2000
0.70	2000	−1.10	1000
0.65	2000	−1.20	1000
0.60	2000	−1.30	1000
0.55	2000	−1.40	1000
0.50	2000	−1.50	1000
0.45	2000	−1.60	1000
0.40	2000	−1.70	1000
0.35	2000	−1.80	1000
0.30	2000	−1.90	1000
0.25	3000	−2.00	1000
0.20	3000	−2.10	1000
0.15	3000	−2.20	1000
0.10	4000	−2.30	1000
0.05	4000	−2.40	1000*
0.00	4000		

* *not* used for symmetric POPC

Table S4: Distribution of umbrella sampling windows along the collective variable with biasing force constants used for atomistic simulations of lipid flip-flop. Reference distances are in nm, force constants in kJ mol^{-1} nm^{-2}.

Reference distance	Force constant	Reference distance	Force constant
2.30	1000	−0.03	2000^R
2.20	1000	−0.06	2000^R
2.10	1000	−0.09	2000^R
2.00	1000	−0.12	2000^R
1.90	1000	−0.15	2000^R
1.80	1000	−0.18	2000^R
1.70	1000	−0.21	2000^R
1.60	1000	−0.25	2000^R
1.50	1000	−0.30	1000
1.40	1000	−0.40	1000
1.30	1000	−0.50	1000
1.20	1000	−0.60	1000
1.10	1000	−0.70	1000
1.00	1000	−0.80	1000
0.90	1000	−0.90	1000
0.80	1000	−1.00	1000
0.70	1000	−1.10	1000
0.60	1000	−1.20	1000
0.50	1000	−1.30	1000
0.40	1000	−1.40	1000
0.30	1000	−1.50	1000
0.25	2000	−1.60	1000
0.21	2000^R	−1.70	1000
0.18	2000^R	−1.80	1000
0.15	2000^R	−1.90	1000
0.12	2000^R	−2.00	1000
0.09	2000^R	−2.10	1000
0.06	2000^R	−2.20	1000
0.03	2000^R	−2.30	1000
0.00	2000^R		

^{R} windows where Hamiltonian replica exchange was applied

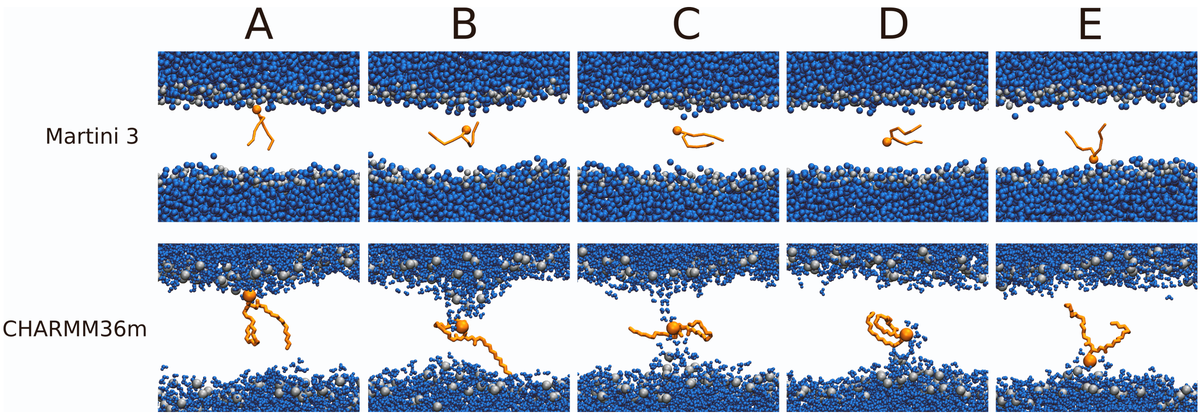

Figure S4: Representative simulation snapshots showing a POPC lipid translocating across a pure POPC membrane, taken from umbrella sampling windows employing the Martini 3 force field (upper row) or CHARMM36m force field (lower row). The snapshots are centered on the headgroup of the translocating lipid. This lipid is highlighted in orange, while only headgroups are shown for other lipids (gray beads). Water molecules are represented by blue beads. A particular emphasis is placed on configurations where the lipid is near the membrane center (panels B–D). In the Martini 3 simulations, there are no significant water defects, membrane disruptions, or water channels, but in the atomistic force field, large water defects are observed. The presence of the lipid at the membrane's center can lead to the formation of water defects from either the upper or the lower leaflet, or from both simultaneously, creating a water channel. The application of Hamiltonian replica exchange to atomistic umbrella sampling windows, where the lipid is near the membrane center, enhances the sampling of these distinct disrupted membrane structures.

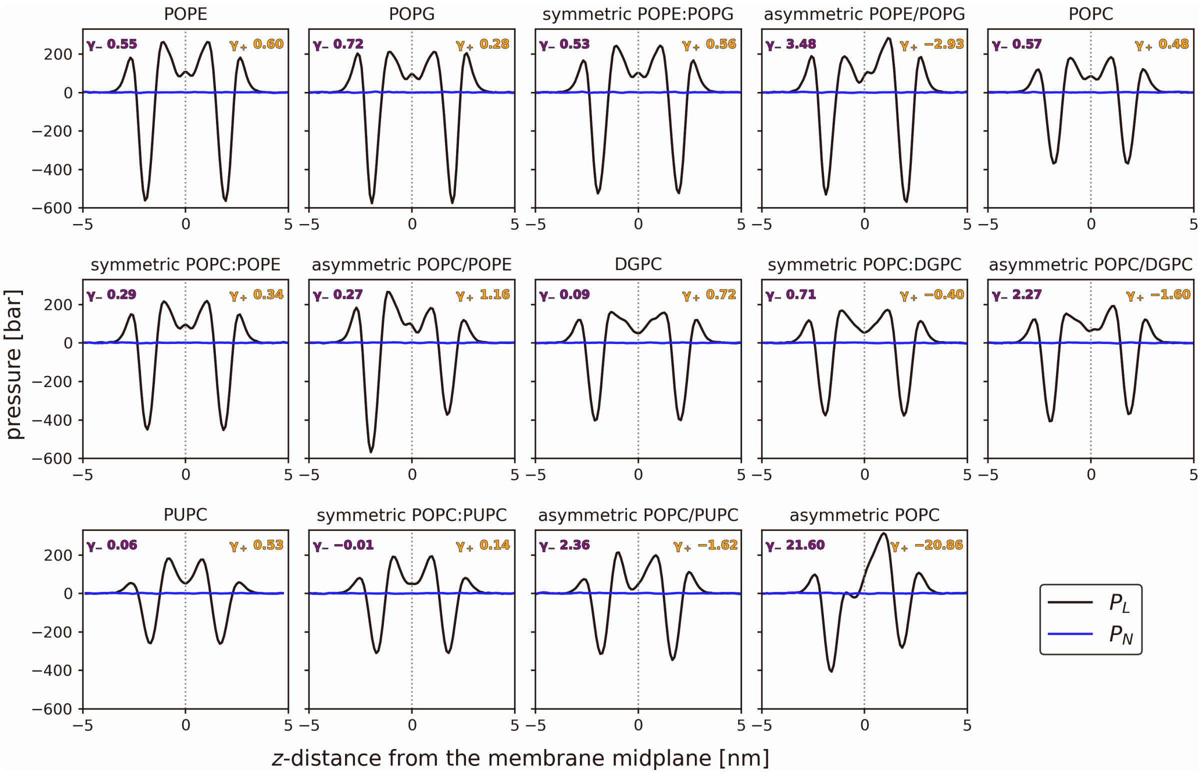

Figure S5: Lateral (P_L) and normal (P_N) pressure profiles along the membrane normal calculated for each peptide-free Martini membrane. Tension of the upper (γ_+, orange) and lower (γ_-, purple) leaflets are presented in units of mN/m. For details concerning the calculation procedure, refer to the Methods section. The calculation error was estimated from the symmetric membranes to be below 1 mN/m.

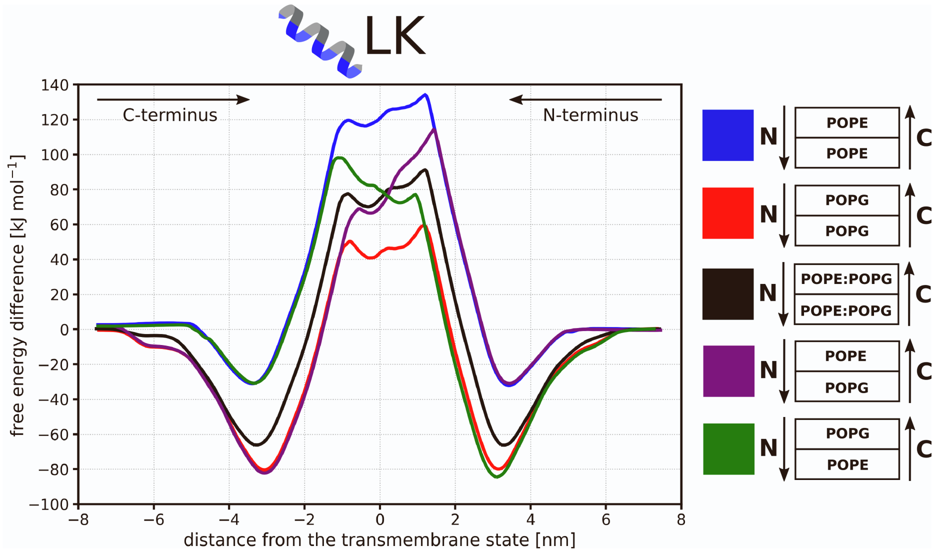

Figure S6: Free energy profiles of the LK peptide translocating through symmetric and asymmetric POPE/POPG membranes (blue = pure POPE, red = pure POPG, black = symmetric POPE:POPG 1:1, purple = asymmetric POPE/POPG, green = asymmetric POPG/POPE). The calculation error is below 5 kJ mol^{-1}. See Figure S7 for the free energy profiles of the individual translocation subprocesses and convergence of the calculations.

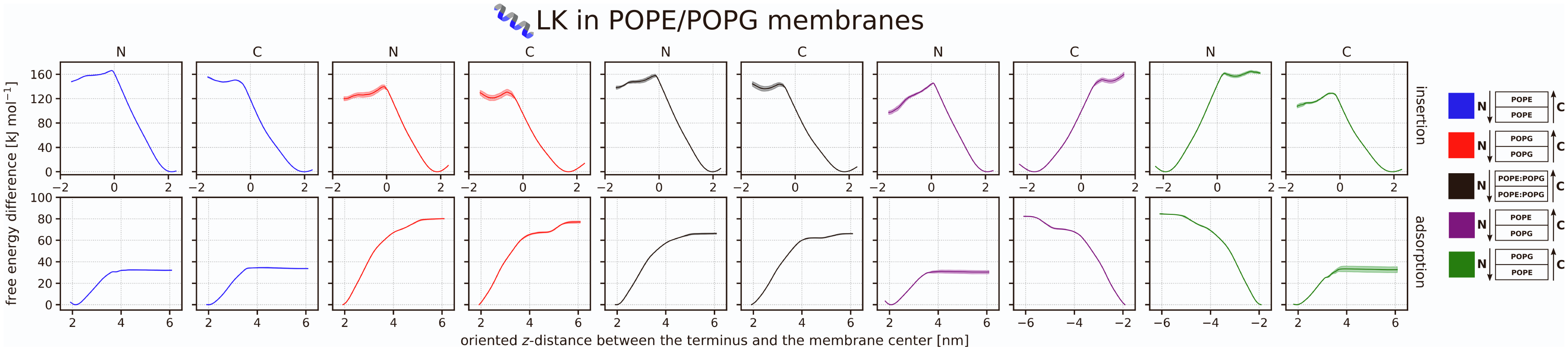

Figure S7: Free energy profiles for individual subprocesses of the LK peptide translocating through both symmetric and asymmetric POPE/POPG membranes. The full translocation profile is constructed by aligning and joining the individual subprocesses calculated for the same system, sharing the color. The color scheme is consistent with Figure S6. The first row presents the free energy profiles of peptide insertion, whereas the second row shows the free energy profiles of peptide adsorption. The columns labeled “N” and “C” correspond to the insertion/adsorption of the peptide’s N-terminus and C-terminus, respectively. Shading depicts the convergence of the calculation as it corresponds to the difference between the free energy profiles calculated from the initial half and the latter half of the production phase of the umbrella sampling simulation.

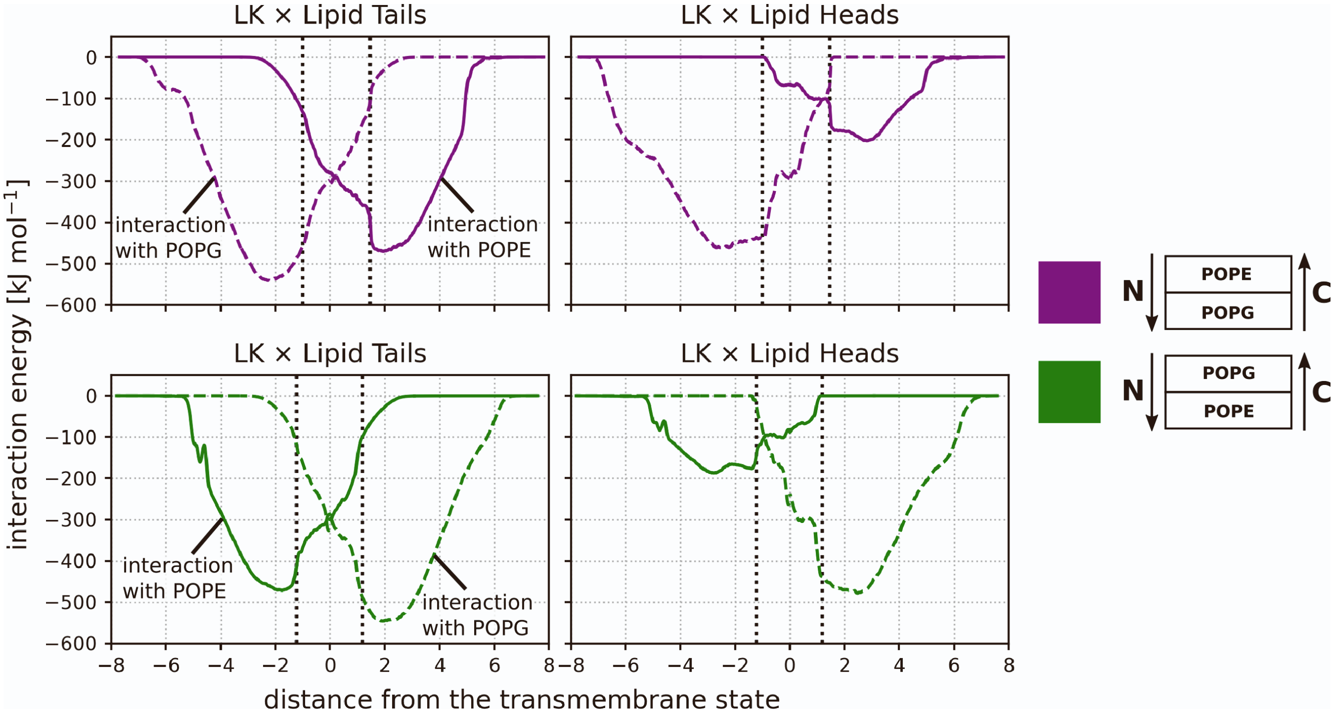

Figure S8: Interaction energy of the LK peptide translocating through asymmetric POPE/POPG membranes with phospholipid tails (left column) and heads (right column). The interaction energies were calculated from all umbrella sampling windows. Purple lines correspond to system in which the peptide translocated through POPE/POPG membrane, while green lines correspond to system in which the peptide translocated through POPG/POPE membrane. Full lines correspond to interaction with POPE lipids while dashed lines correspond to interaction with POPG lipids. Vertical dotted lines show approximate positions where the translocation profile for the asymmetric membrane differs from that for the symmetric membrane.

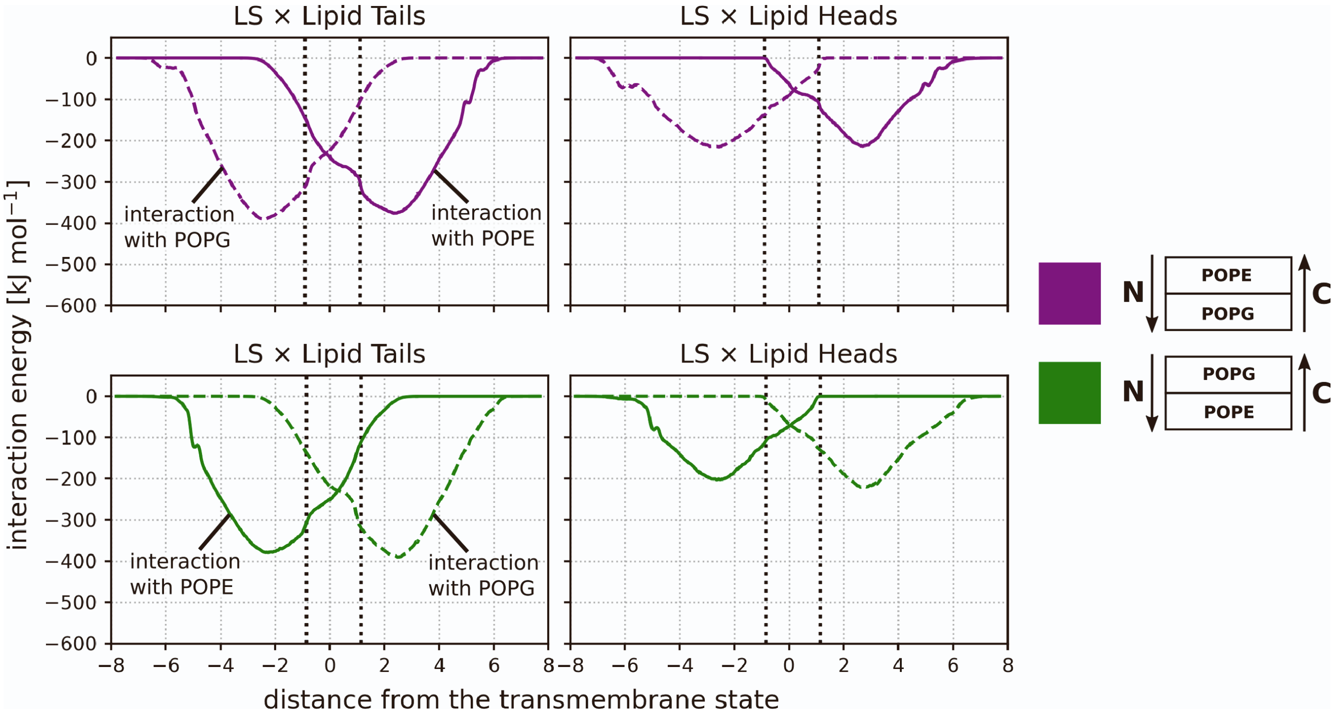

Figure S9: Interaction energy of the LS peptide translocating through asymmetric POPE/POPG membranes with phospholipid tails (left column) and heads (right column). The interaction energies were calculated from all umbrella sampling windows. Purple lines correspond to system in which the peptide translocated through POPE/POPG membrane, while green lines correspond to system in which the peptide translocated through POPG/POPE membrane. Full lines correspond to interaction with POPE lipids while dashed lines correspond to interaction with POPG lipids. Vertical dotted lines show approximate positions where the translocation profile for the asymmetric membrane differs from that for the symmetric membrane.

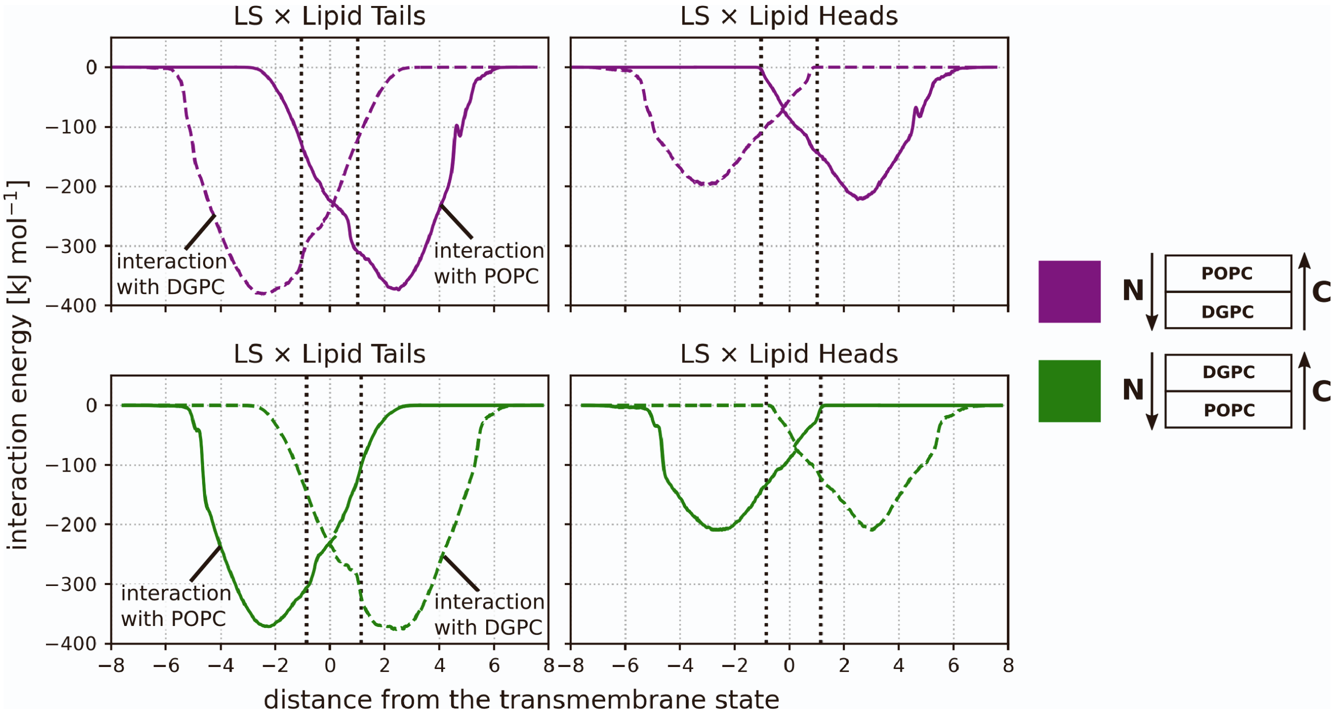

Figure S10: Interaction energy of the LS peptide translocating through asymmetric POPC/DGPC membranes with phospholipid tails (left column) and heads (right column). The interaction energies were calculated from all umbrella sampling windows. Purple lines correspond to system in which the peptide translocated through POPC/DGPC membrane, while green lines correspond to system in which the peptide translocated through DGPC/POPC membrane. Full lines correspond to interaction with POPC lipids while dashed lines correspond to interaction with DGPC lipids. Vertical dotted lines show approximate positions where the translocation profile for the asymmetric membrane differs from that for the symmetric membrane.

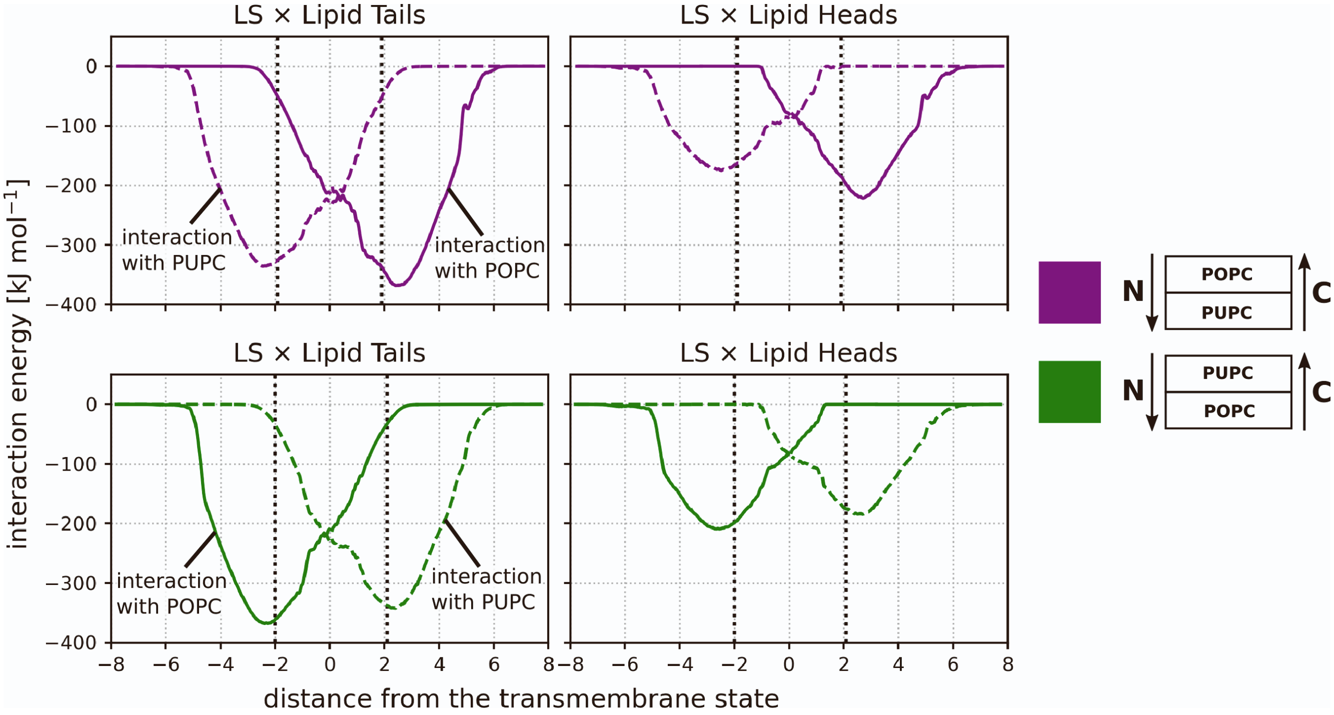

Figure S11: Interaction energy of the LS peptide translocating through asymmetric POPC/PUPC membranes with phospholipid tails (left column) and heads (right column). The interaction energies were calculated from all umbrella sampling windows. Purple lines correspond to system in which the peptide translocated through POPC/PUPC membrane, while green lines correspond to system in which the peptide translocated through PUPC/POPC membrane. Full lines correspond to interaction with POPC lipids while dashed lines correspond to interaction with PUPC lipids. Vertical dotted lines show approximate positions where the translocation profile for the asymmetric membrane differs from that for the symmetric membrane.

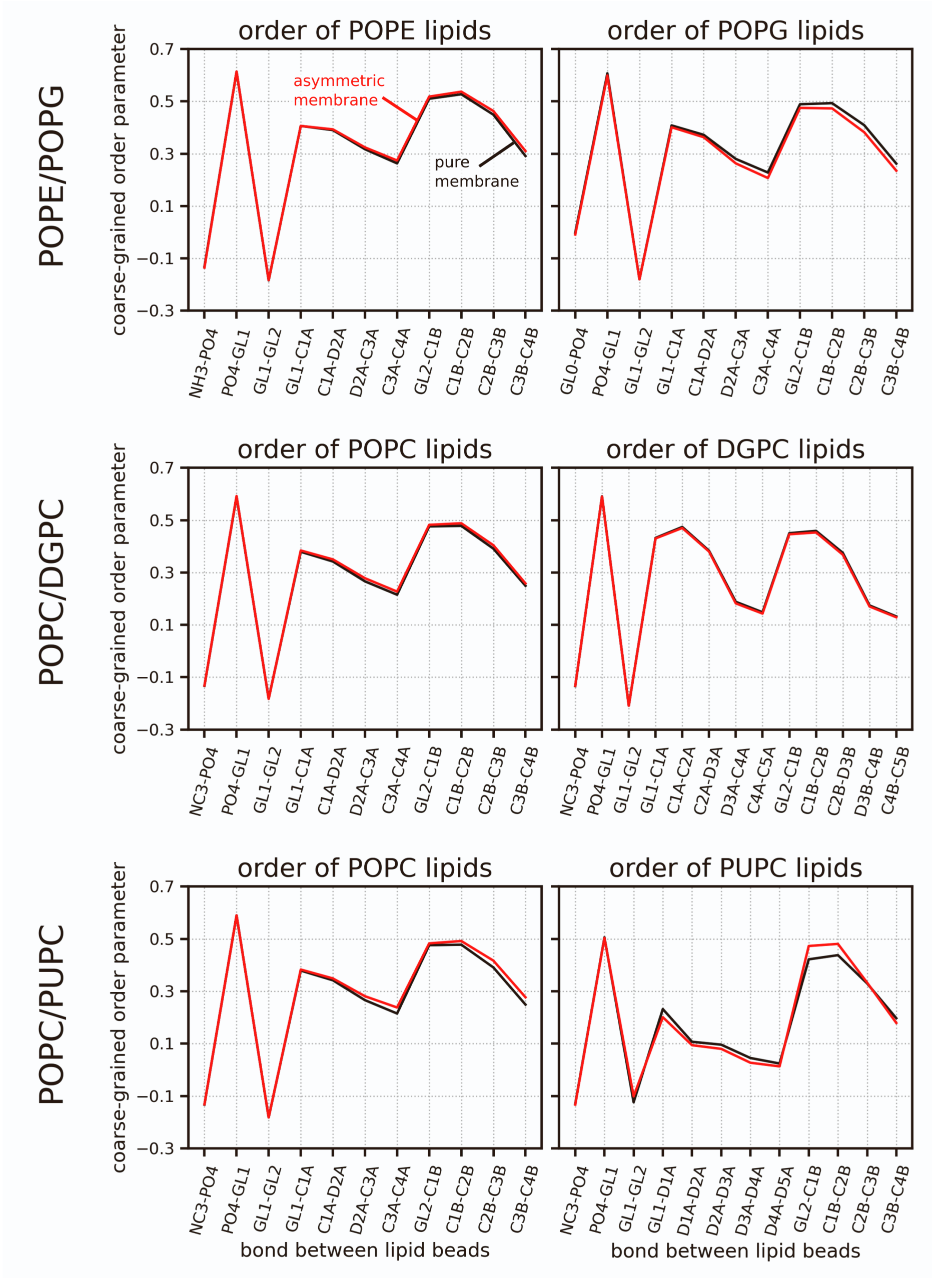

Figure S12: Coarse-grained order parameters calculated in symmetric and asymmetric membranes without the presence of a peptide. First row corresponds to membranes composed of POPE and POPG lipids. Second row corresponds to membranes composed of POPC and DGPC lipids. Third row corresponds to membranes composed of POPC and PUPC lipids. Black lines show order parameters calculated in one-component symmetric membranes composed of the concerned lipid type. Red lines show order parameters of the concerned lipid type calculated in an asymmetric membrane. The magnitude of the difference between the black and red line reflects how much the studied lipids are affected by the presence of the other leaflet, which is composed of a different lipid type, in the asymmetric membrane. Order parameters were calculated using the `order` tool available from doi.org/10.5281/zenodo.8369479.

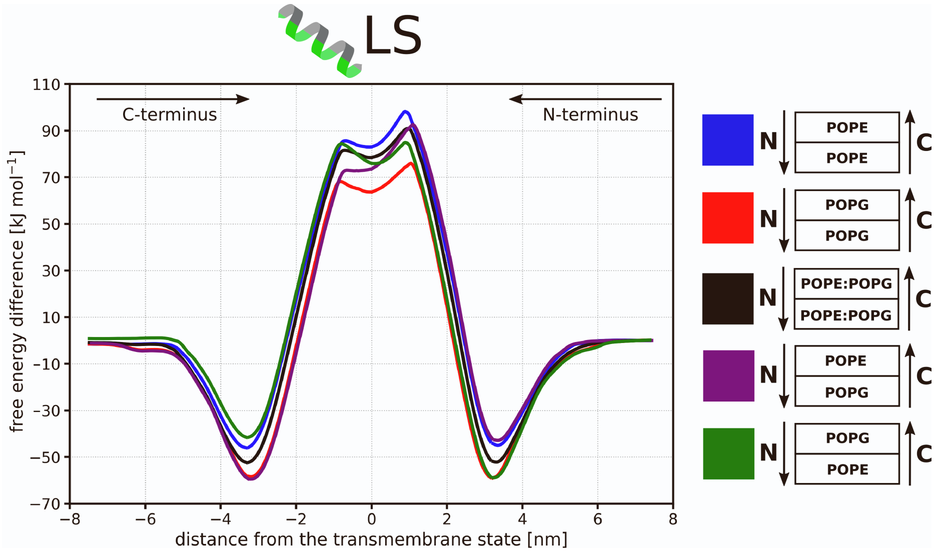

Figure S13: Free energy profiles of the LS peptide translocating through symmetric and asymmetric POPE/POPG membranes (blue = pure POPE, red = pure POPG, black = symmetric POPE:POPG 1:1, purple = asymmetric POPE/POPG, green = asymmetric POPG/POPE). The calculation error is below 5 kJ mol^{-1}. See Figure S16 for free energy profiles of the individual translocation subprocesses and convergence of the calculations.

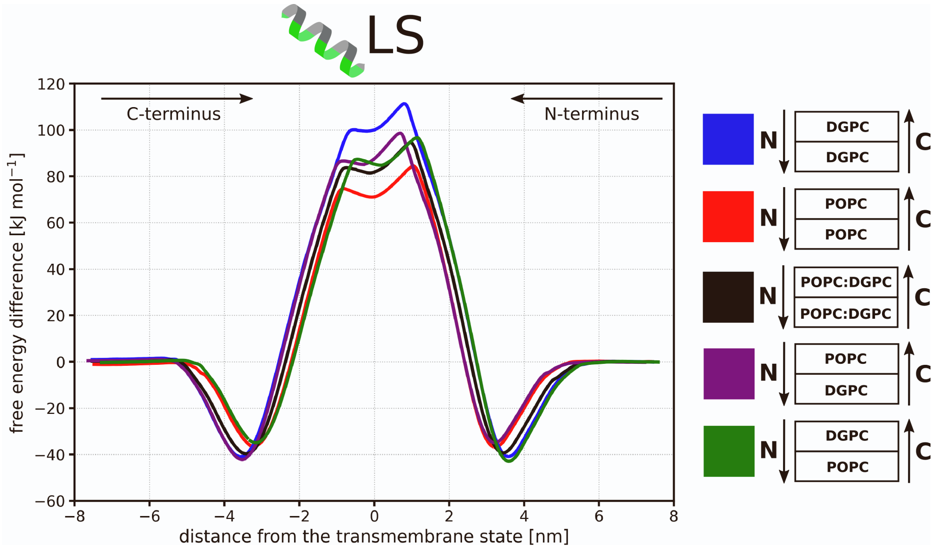

Figure S14: Free energy profiles of the LS peptide translocating through symmetric and asymmetric POPC/DGPC membranes (blue = pure DGPC, red = pure POPC, black = symmetric POPC:DGPC 1:1, purple = asymmetric POPC/DGPC, green = asymmetric DGPC/POPC). The calculation error is below 5 kJ mol^{-1}. See Figure S17 for free energy profiles of the individual translocation subprocesses and convergence of the calculations.

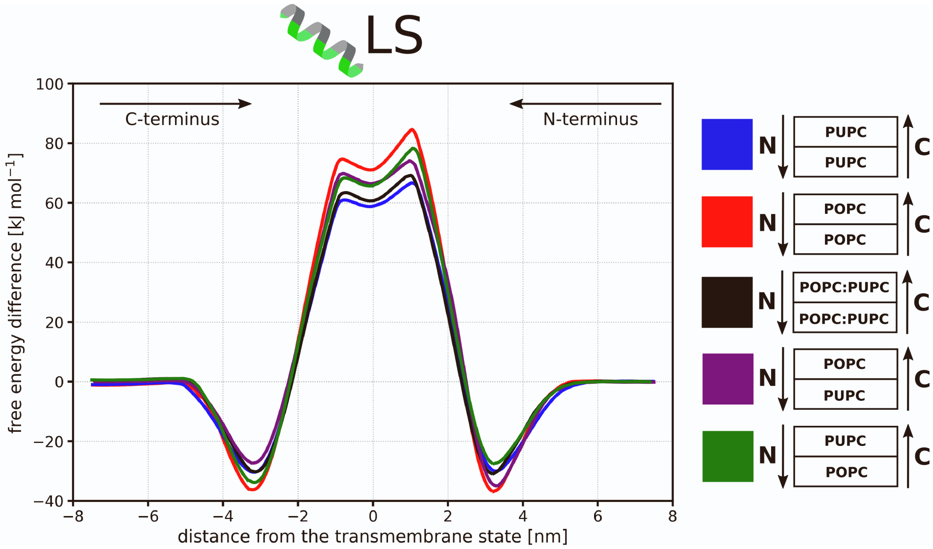

Figure S15: Free energy profiles of the LS peptide translocating through symmetric and asymmetric POPC/PUPC membranes (blue = pure PUPC, red = pure POPC, black = symmetric POPC:PUPC 1:1, purple = asymmetric POPC/PUPC, green = asymmetric PUPC/POPC). The calculation error is below 5 kJ mol^{-1}. See Figure S18 for free energy profiles of the individual translocation subprocesses and convergence of the calculations.

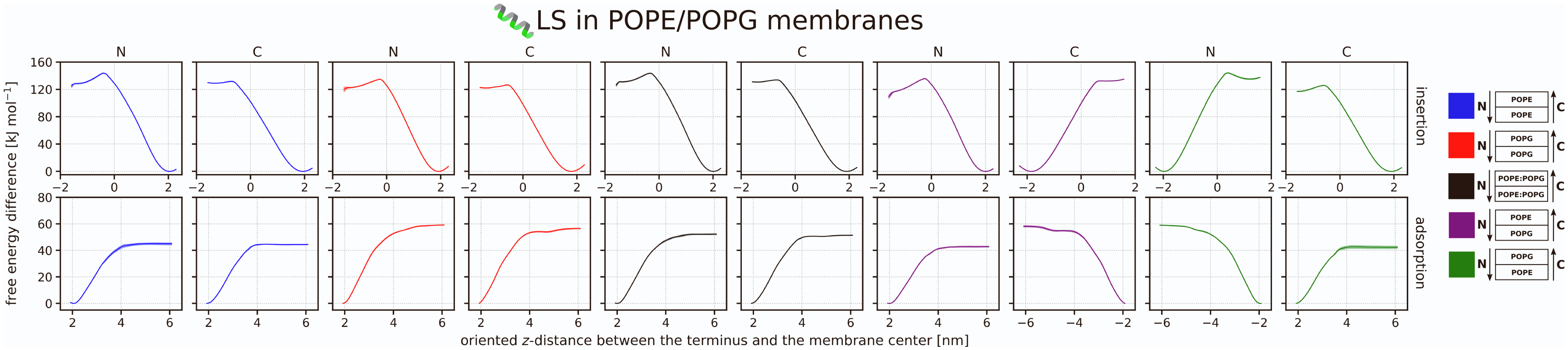

Figure S16: Free energy profiles for individual subprocesses of the LS peptide translocating through both symmetric and asymmetric POPE/POPG membranes. The full translocation profile is constructed by aligning and joining the individual subprocesses calculated for the same system, sharing the color. The color scheme is consistent with that in Figure S13. The first row presents the free energy profiles of peptide insertion, whereas the second row shows the free energy profiles of peptide adsorption. The columns labeled “N” and “C” correspond to the insertion/adsorption of the peptide’s N-terminus and C-terminus, respectively. Shading depicts the convergence of the calculation as it corresponds to the difference between the free energy profiles calculated from the initial half and the latter half of the production phase of the umbrella sampling simulation.

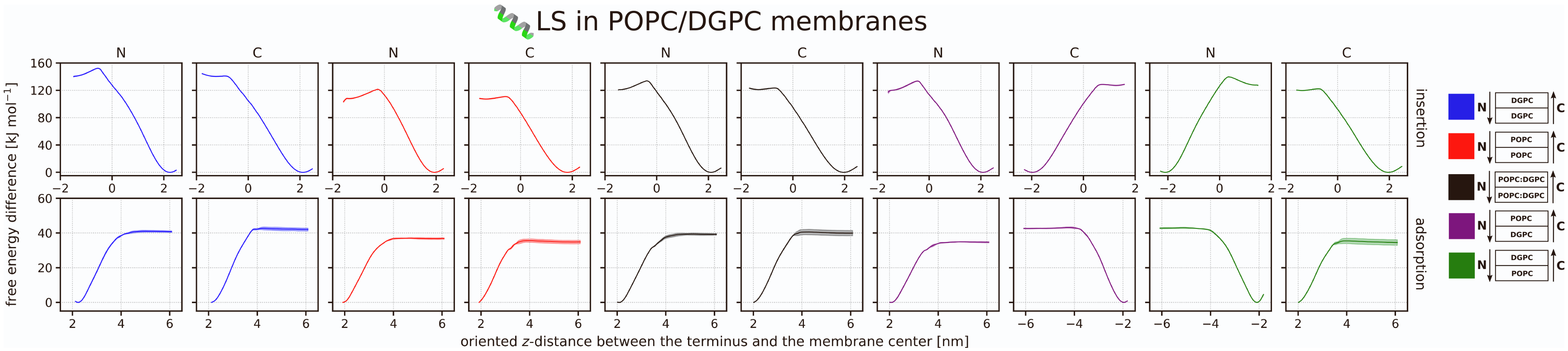

Figure S17: Free energy profiles for individual subprocesses of the LS peptide translocating through both symmetric and asymmetric POPC/DGPC membranes. The full translocation profile is constructed by aligning and joining the individual subprocesses calculated for the same system, sharing the color. The color scheme is consistent with that in Figure S14. The first row presents the free energy profiles of peptide insertion, whereas the second row shows the free energy profiles of peptide adsorption. The columns labeled “N” and “C” correspond to the insertion/adsorption of the peptide’s N-terminus and C-terminus, respectively. Shading depicts the convergence of the calculation as it corresponds to the difference between the free energy profiles calculated from the initial half and the latter half of the production phase of the umbrella sampling simulation.

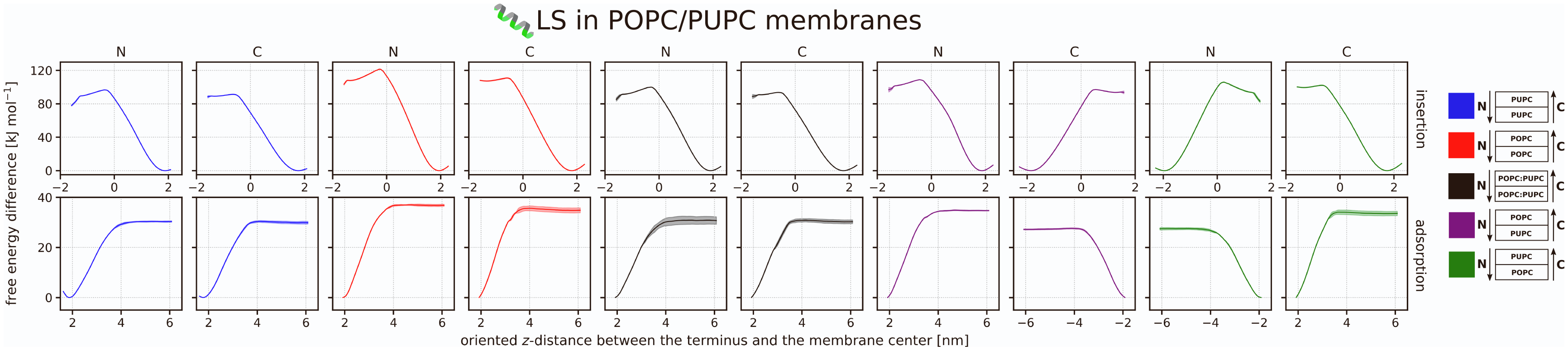

Figure S18: Free energy profiles for individual subprocesses of the LS peptide translocating through both symmetric and asymmetric POPC/PUPC membranes. The full translocation profile is constructed by aligning and joining the individual subprocesses calculated for the same system, sharing the color. The color scheme is consistent with that in Figure S15. The first row presents the free energy profiles of peptide insertion, whereas the second row shows the free energy profiles of peptide adsorption. The columns labeled “N” and “C” correspond to the insertion/adsorption of the peptide’s N-terminus and C-terminus, respectively. Shading depicts the convergence of the calculation as it corresponds to the difference between the free energy profiles calculated from the initial half and the latter half of the production phase of the umbrella sampling simulation.

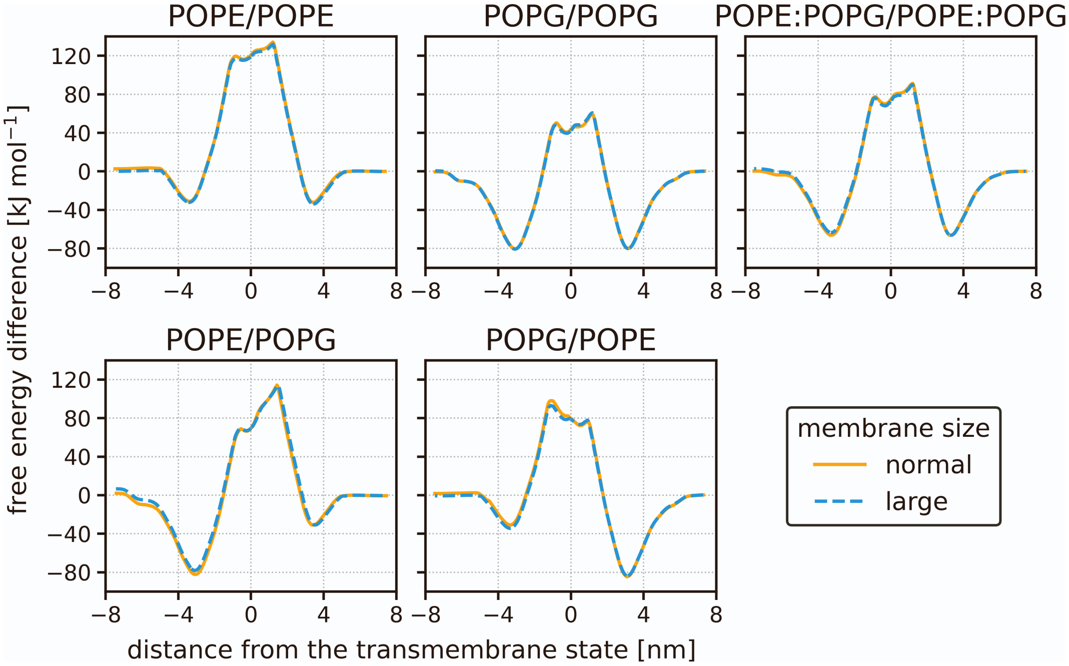

Figure S19: Free energy profiles of the LK peptide translocating through symmetric and asymmetric POPE/POPG membranes. The profiles include those for membranes composed of approximately 290 lipid molecules (normal membrane size, orange) and for larger membranes composed of roughly 970 lipid molecules (large membrane size, blue). No systematic differences were observed between the profiles calculated in normal-sized and larger membranes. This suggests that the behavior described in the main text is not an artifact of the periodic system size.

Table S5: Free energy differences for key stages in the translocation profiles of the peptides LK and LS across various symmetric and asymmetric membranes. The displayed free energy values are given relative to the peptide in solution [kJ mol^{-1}]. The error was estimated to be below 5 kJ mol^{-1}. ΔG_{AN} and ΔG_{AC} represent the free energies of the peptide's adsorbed states on either side of the membrane. ΔG_{IN} and ΔG_{IC} denote the maximum free energies when the peptide inserts its N- or C-terminus into the membrane. ΔG_{TM} corresponds to the peptide's free energy in its transmembrane state. $\Delta\Delta G_{BN}$ and $\Delta\Delta G_{BC}$ represent the translocation barriers when the peptide inserts using its N-terminus (BN) or C-terminus (BC) first. These barriers are computed by subtracting the adsorbed state's free energy (ΔG_{AN} for BN and ΔG_{AC} for BC) from the profile's maximum free energy. A higher translocation barrier signifies a more difficult translocation. See Figure S20 for graphical explanation of the free energy values of interest.

peptide	membrane	ΔG_{AN}	ΔG_{AC}	ΔG_{IN}	ΔG_{IC}	ΔG_{TM}	$\Delta\Delta G_{BN}$	$\Delta\Delta G_{BC}$
LK	POPE	−32	−31	134	120	120	166	165
	POPG	−80	−80	60	50	44	140	140
	sym POPE:POPG	−66	−66	91	77	74	157	157
	asym POPE/POPG	−31	−82	114	69	69	145	196
	asym POPG/POPE	−84	−31	80	98	80	182	129
LS	POPE	−45	−46	98	86	83	143	144
	POPG	−59	−58	76	68	74	135	134
	sym POPE:POPG	−52	−52	91	82	78	143	143
	asym POPE/POPG	−43	−59	93	74	74	136	152
	asym POPG/POPE	−59	−42	85	84	76	144	127
LS	DGPC	−41	−41	111	100	100	152	152
	POPC	−37	−36	85	75	71	122	121
	sym POPC:DGPC	−39	−40	95	84	82	134	135
	asym POPC/DGPC	−35	−42	99	87	88	134	141
	asym DGPC/POPC	−43	−35	97	87	85	140	132
LS	PUPC	−30	−30	67	61	59	97	97
	POPC	−37	−36	85	75	71	122	121
	sym POPC:PUPC	−31	−30	69	63	61	100	99
	asym POPC/PUPC	−35	−27	74	70	66	109	101
	asym PUPC/POPC	−28	−34	78	68	66	106	112

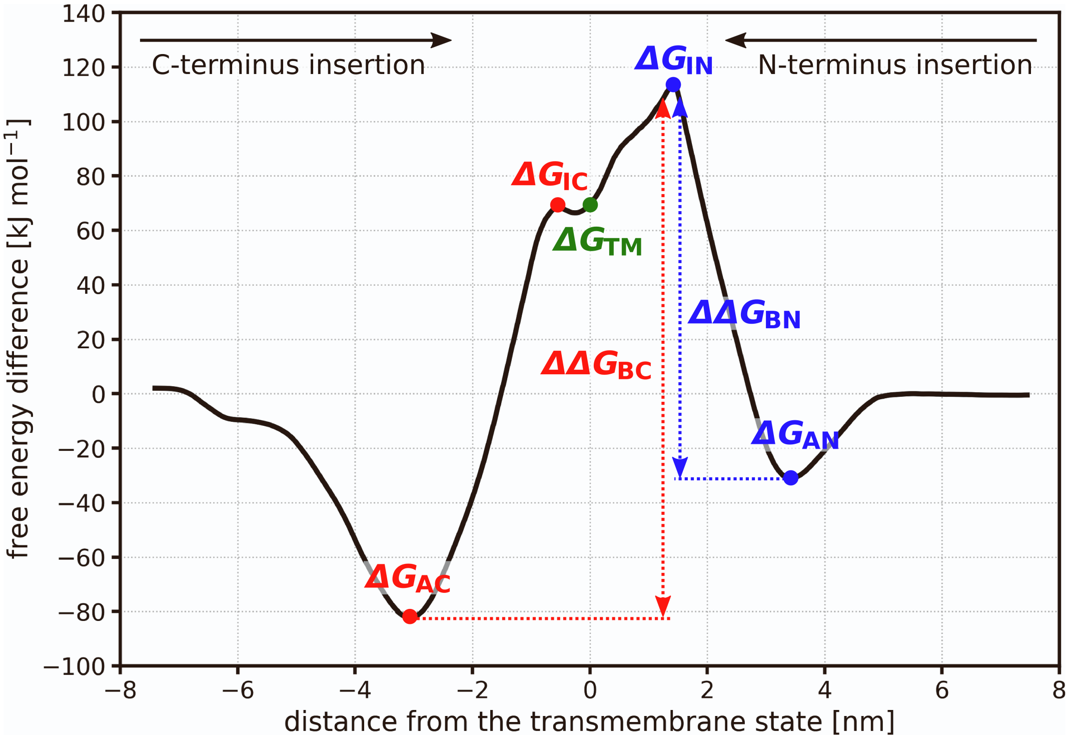

Figure S20: Free energy profile of the LK peptide translocating through asymmetric POPE/POPG membrane with important points in the profile highlighted. ΔG_{AN} and ΔG_{AC} represent the free energies of the adsorbed states of the peptide on either side of the membrane. ΔG_{IN} and ΔG_{IC} denote the free energy maxima when the peptide inserts its N- or C-terminus into the membrane. ΔG_{TM} represents the free energy of the peptide in its transmembrane state. $\Delta\Delta G_{\mathrm{BN}}$ and $\Delta\Delta G_{\mathrm{BC}}$ correspond to the translocation barriers when the peptide inserts with its N-terminus (BN) or C-terminus (BC) first. The translocation barrier is determined by the difference between the profile's free energy maximum and the free energy of the relevant adsorbed state. In this case, $\Delta\Delta G_{\mathrm{BN}} = \Delta G_{\mathrm{IN}} - \Delta G_{\mathrm{AN}}$ and $\Delta\Delta G_{\mathrm{BC}} = \Delta G_{\mathrm{IN}} - \Delta G_{\mathrm{AC}}$.

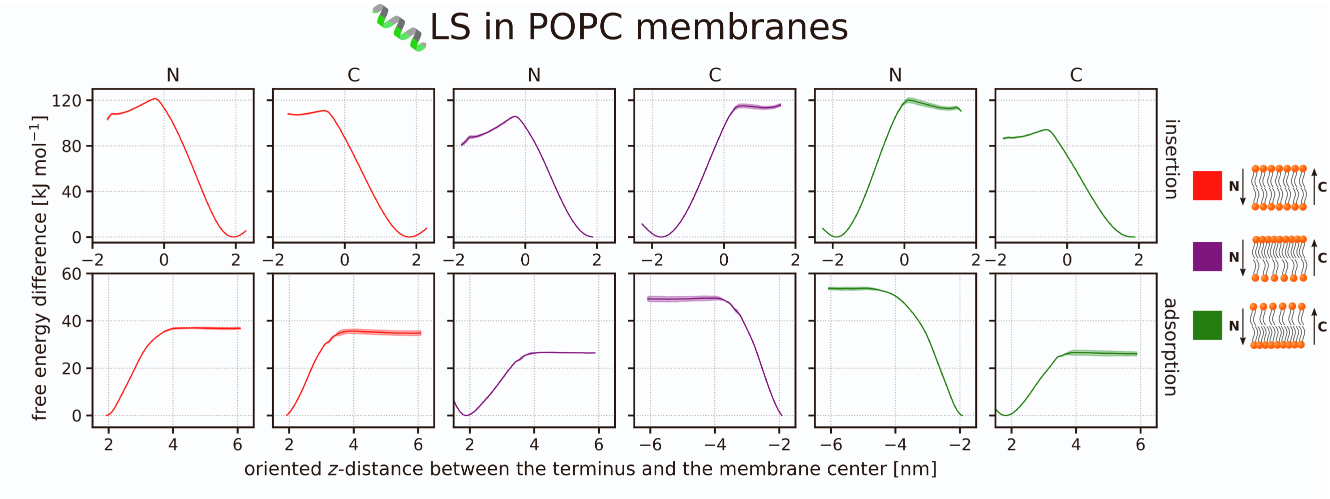

Figure S21: Free energy profiles for individual subprocesses of the LS peptide translocating through both symmetric and asymmetric POPC membranes. The full translocation profile is constructed by aligning and joining the individual subprocesses calculated for the same system, sharing the color. The color scheme is consistent with that in Figure 3. The first row presents the free energy profiles of peptide insertion, whereas the second row shows the free energy profiles of peptide adsorption. The columns labeled "N" and "C" correspond to the insertion/adsorption of the peptide's N-terminus and C-terminus, respectively. Shading depicts the convergence of the calculation as it corresponds to the difference between the free energy profiles calculated from the initial half and the latter half of the production phase of the umbrella sampling simulation.

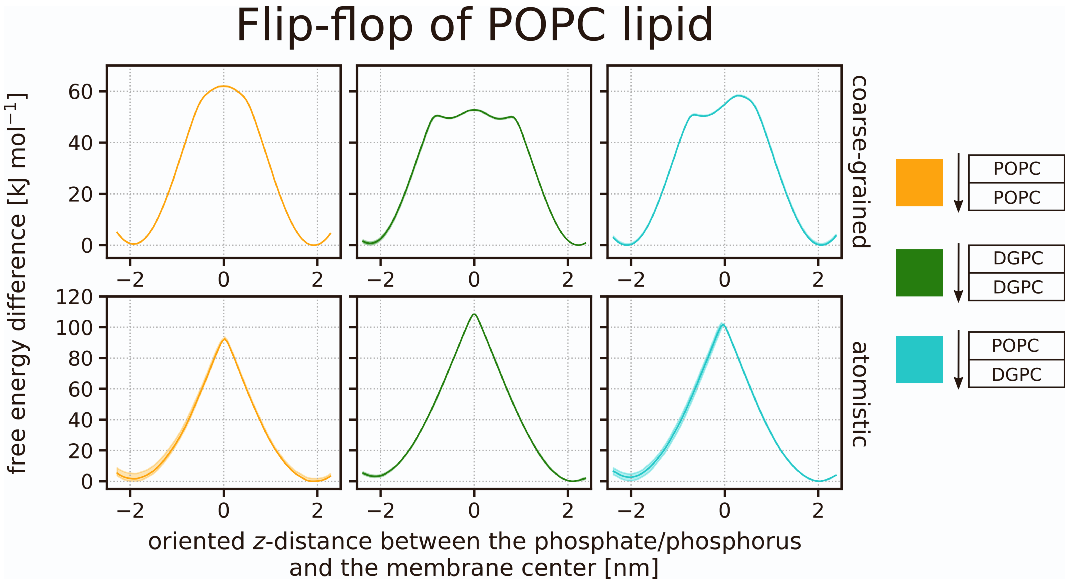

Figure S22: Free energy profiles of POPC lipid translocating through symmetric and asymmetric POPC/DGPC membranes. The color scheme is consistent with that in Figure 4. Shading is used to illustrate the convergence of the calculation. The width of the shaded area corresponds to the difference between the free energy profiles calculated from the initial half and the latter half of the production phase of the umbrella sampling simulation. A more expansive shaded region denotes a greater margin of error.
