# Supplementary material for: Peptide translocation across asymmetric phospholipid membranes
Source: Biophys J. 2024 Feb 15;123(6):693–702. doi: 10.1016/j.bpj.2024.02.006 (PMC10995401; doi:10.1016/j.bpj.2024.02.006)
Supplement: Document S2. Article plus supporting material [file mmc2.pdf]

Biophysical Journal
Article

Biophysical Society

Peptide translocation across asymmetric phospholipid membranes

Ladislav Bartoš[1,2] and Robert Vácha[1,2,3,*]
[1]CEITEC – Central European Institute of Technology, Brno, Czech Republic; [2]National Centre for Biomolecular Research, Faculty of Science, Masaryk University, Brno, Czech Republic; and [3]Department of Condensed Matter Physics, Faculty of Science, Masaryk University, Brno, Czech Republic

ABSTRACT The transport of molecules across cell membranes is vital for proper cell function and effective drug delivery. While most cell membranes naturally possess an asymmetric lipid composition, research on membrane transport predominantly uses symmetric lipid membranes. The permeation through the asymmetric membrane is then calculated as a sum of the inverse permeabilities of leaflets from symmetric bilayers. In this study, we examined how two types of amphiphilic molecules translocate across both asymmetric and symmetric membranes. Using computer simulations with both coarse-grained and atomistic force fields, we calculated the free energy profiles for the passage of model amphiphilic peptides and a lipid across various membranes. Our results consistently demonstrate that while the free energy profiles for asymmetric membranes with a small differential stress concur with symmetric ones in the region of lipid headgroups, the profiles differ around the center of the membrane. In this region, the free energy for the asymmetric membrane transitions between the profiles for two symmetric membranes. In addition, we show that peptide permeability through an asymmetric membrane cannot always be predicted from the permeabilities of the symmetric membranes. This indicates that using symmetric membranes falls short in providing an accurate depiction of peptide translocation across asymmetric membranes.

SIGNIFICANCE Cells are separated from their surroundings by a semipermeable cytoplasmic membrane. Peptides with specific properties are able to spontaneously cross this barrier and act as drugs or drug carriers. Typically, the permeation of these peptides is studied using symmetric model membranes, even though actual cell membranes are usually asymmetric. It is commonly thought that the permeability of an asymmetric membrane can be approximated from the permeabilities of the corresponding symmetric membranes. However, our findings indicate that for some peptides, this assumption does not hold true. Specifically, the permeability of a peptide through an asymmetric membrane can vary considerably and can even be much lower or much higher than the permeability through the related symmetric membranes.

INTRODUCTION

Biological membranes are semipermeable barriers enabling some molecules to spontaneously pass into the cell. While these molecules are usually small and uncharged, it has been documented that larger, amphiphilic molecules, including peptides, can also undergo spontaneous translocation through membranes. Antimicrobial peptides, for instance, can translocate across cell membranes and disrupt intracellular processes, leading to cell death (1,2). Similarly, cell-penetrating peptides are utilized to transport various types of molecular cargo, including drugs, across cell membranes and into the cytosol (3,4). Therefore, understanding the mechanisms of peptide translocation can be utilized in the development of new therapies against infectious diseases.

Many biological membranes are inherently asymmetric, exhibiting different lipid compositions and properties in each leaflet (5–7). Despite the asymmetry, both computational (8–12) and experimental (10,13,14) research has primarily centered on peptide translocation across symmetric membranes, i.e., bilayers with the same lipid composition in both leaflets. The symmetric choice is partly due to the challenges associated with constructing asymmetric bilayers for in vitro studies (15). While there are computational

Submitted November 24, 2023, and accepted for publication February 9, 2024.

*Correspondence: robert.vacha@muni.cz

Editor: Frederick Heberle.

https://doi.org/10.1016/j.bpj.2024.02.006

© 2024 Biophysical Society.

This is an open access article under the CC BY license (http://creativecommons.org/licenses/by/4.0/).

studies involving asymmetric membranes (16–21), they have not specifically investigated the translocation of antimicrobial and cell-penetrating peptides. As a result, our knowledge of peptide translocation across asymmetric membranes is limited, and it remains uncertain whether findings from symmetric membrane studies can be reliably applied to their asymmetric counterparts.

Here, we address this knowledge gap by studying peptide translocation across asymmetric membranes using coarse-grained molecular dynamics simulations with free energy calculations. Our findings indicate that the rate at which a peptide translocates through an asymmetric membrane often cannot be accurately inferred from the rates observed in symmetric membranes composed of the same lipids as the individual leaflets of the asymmetric membrane. For asymmetric membranes with a small differential stress, we identify specific translocation regions that resemble the behavior seen in symmetric membranes, as well as regions that exhibit unique characteristics. Additionally, by employing both coarse-grained and atomistic free energy calculations, we investigate lipid flip-flop in asymmetric membranes, which demonstrates trends in the free energy profiles analogous to those of the peptides.

MATERIALS AND METHODS

Coarse-grained peptide translocation simulations

All peptide translocation simulations were performed using the Gromacs simulation package v2021.4 (22) with a coarse-grained Martini force field 3.0 (23).

We used two different model amphiphilic peptides: LS, a peptide with the sequence LSSLLSLLSSLLSLLSSLLSL-NH2, and LK, a positively charged peptide with the sequence LKKLLKLLKKLLKLLKKLLKL-NH2. The LS peptide was chosen as a representative amphiphilic helix with a hydrophobic and a hydrophilic patch. Similar peptides were also used in our previous research (9,24). The positively charged LK peptide was selected as a representative of antimicrobial and cell-penetrating peptides, which usually carry a large positive charge. Both peptides had a positively charged N-terminus and a neutral (amidated) C-terminus. Peptides and proteins were constructed using Modeller v9.11 (25) and then coarse grained using the martinize2 script (https://github.com/marrink-lab/vermouth-martinize). Both peptides were restrained to stay in an α-helical conformation. Each coarse-grained peptide was minimized in vacuum using the steepest-descent algorithm with maximum force tolerance of 100 kJ mol^{-1} nm^{-1}.

We used 14 symmetric and asymmetric membranes composed of 1-palmitoyl-2-oleoyl-*sn*-glycero-3-phosphocholine (POPC; C16:0/C18:1 PC), 1-palmitoyl-2-oleoyl-*sn*-glycero-3-phosphoethanolamine (POPE; C16:0/C18:1 PE), 1-palmitoyl-2-oleoyl-*sn*-glycero-3-phosphoglycerol (POPG; C16:0/C18:1 PG), 1,2-di-(11Z-eicosenoyl)-*sn*-glycero-3-phosphocholine (DGPC; di-C20:1 PC), and 1-palmitoyl-2-(4Z, 7Z, 10Z, 13Z, 16Z, 19Z-docosahexaenoyl)-*sn*-glycero-3-phosphocholine (PUPC; C16:0/C22:6 PC). All of the used lipids mixed with each other when simulated at 310 K using the Martini 3 force field and formed homogeneous bilayers, i.e., no phase separation was observed in any of the simulated membranes.

Membranes were constructed using the insane script (https://github.com/Tsjerk/Insanegithub.com/Tsjerk/Insane). In case of asymmetric two-component membranes, we further manually removed several lipids from one of the leaflets to achieve matching leaflet surface areas and small differential stress in the membrane. Single-component symmetric membranes consisted of 144 lipids in each membrane leaflet (for a total of 288 lipids in the entire membrane). Two-component symmetric membranes consisted of 72 lipids of each concerned lipid type in each leaflet (for a total of 288 lipids in the entire membrane). The asymmetric POPE/POPG membrane was composed of 144 POPE lipids in the upper leaflet and 135 POPG lipids in the lower leaflet, the asymmetric POPC/DGPC membrane consisted of 144 POPC lipids in the upper leaflet and 138 DGPC lipids in the lower leaflet, and the asymmetric POPC/PUPC membrane consisted of 144 POPC lipids in the upper leaflet and 120 PUPC lipids in the lower leaflet. The asymmetric single-component POPC membrane contained 144 POPC lipids in the upper leaflet and 106 POPC lipids in the lower leaflet. For insertion simulations, each system had an approximate size of $10 \times 10 \times 11$ nm and was solvated with roughly 5500 water beads. For adsorption simulations, the systems were larger at $10 \times 10 \times 17$ nm and solvated with roughly 10,000 water beads.

We have also prepared several larger membranes composed of POPE and/or POPG. Large POPE and POPG membranes contained 484 lipids in each leaflet, and large symmetric POPE:POPG consisted of 242 POPE and 242 POPG lipids in each leaflet. Each of these membranes consisted of 968 lipid molecules in total. The large asymmetric POPE/POPG membrane was composed of 515 POPE lipids in one leaflet and 484 POPG lipids in the other leaflet. For insertion simulations, each large system had an approximate size of $18 \times 18 \times 9$ nm and was solvated with roughly 14,000 water beads. For adsorption simulations, each large system had an approximate size of $18 \times 18 \times 17$ nm and was solvated with roughly 35,000 water beads.

The potential energy of each membrane was minimized without a peptide using the steepest-descent algorithm and force tolerance of 100 kJ mol^{-1} nm^{-1} and was equilibrated in five stages of different simulation lengths and time steps: 1) $dt = 2$ fs, $t = 0.5$ ns; 2) $dt = 5$ fs, $t = 1.25$ ns; 3) $dt = 10$ fs, $t = 1$ ns; 4) $dt = 20$ fs, $t = 30$ ns; and 5) $dt = 20$ fs, $t = 1000$ ns. In stages 1–4, the Berendsen barostat (26) was employed, which was replaced with the Parrinello-Rahman barostat (27,28) in stage 5. All other simulation settings remained the same during the individual stages of equilibration as well as in all the following simulations. All simulations were performed in NPT ensemble with temperature maintained at 310 K using a stochastic velocity rescaling thermostat (29) with a coupling constant of 1 ps. Water with ions and membrane with peptide (if present) were coupled to two separate thermal baths. Pressure was kept at 1 bar using either the Berendsen (26) or Parrinello-Rahman (27,28) barostat (see above) with a coupling constant of 12 ps. Semi-isotropic pressure coupling was employed to independently scale the simulation box in the xy plane and on the z axis with a compressibility of 3×10^{-4} bar^{-1}. The leap-frog algorithm was used to integrate the equations of motion. For nonbonded interactions, we used the cutoff distance of 1.1 nm. The van der Waals potential was shifted to zero at the cutoff distance. The relative dielectric constant was set to 15.

All simulations with the large membranes composed of POPE and/or POPG lipids (see above) were conducted with neighbor list parameters modified according to Kim et al. (30) to avoid errors in the instantaneous pressure tensor. Verlet-buffer-tolerance was set to -1; nstlist, nsttcouple, and nstpcouple were all set to 20; and rlist was 1.35.

After membrane equilibration, a peptide was placed onto the membrane surface in an orientation parallel with the membrane plane. Subsequently, we added NaCl ions at a concentration of 0.154 mol dm^{-3} (with an excess of ions to neutralize the system). The potential energy of the system was then minimized with the same settings as for membrane minimization. Stages 1–4 of system equilibration were the same as stages 1–4 of membrane equilibration, while stage 5 was shortened to 100 ns, and an additional stage ($dt = 20$ fs, $t = 15$ ns) was added between stages 4 and 5. In stages 1–4, all backbone beads of peptide were restrained to their initial positions using a harmonic potential with a force constant of 1000 kJ mol^{-1} nm^{-2}. In the remaining two stages, peptide was restrained to stay in the adsorbed state using two harmonic potentials applied to their N and C termini (first and last three backbone beads of the peptide).

The umbrella sampling method (31,32) was used to enhance the sampling of the configuration space. Similarly to our previous studies (8,9), we have divided the translocation process into eight separate subprocesses: N-terminus insertion from the upper and lower leaflets, C-terminus insertion from the upper and lower leaflets, N-terminus adsorption onto the upper and lower leaflets, and C-terminus adsorption onto the upper and lower leaflets. In the case of symmetric membranes, it does not matter from which side of the membrane the peptide inserts into the membrane or onto which membrane leaflet it adsorbs. Therefore, peptide translocation across symmetric membranes can be completely characterized by calculating the free energy of just four individual subprocesses: N-terminus adsorption, N-terminus insertion, C-terminus insertion, and C-terminus adsorption.

To describe the individual insertions/adsorptions, we employed a collective variable that was the oriented distance between the center of mass of the peptide terminus (first—N—or last—C—three backbone beads of the peptide) and local membrane center of mass on z axis. The local membrane center of mass was calculated from the positions of lipid beads localized in a cylinder with the radius of 2.0 nm and its principal axis going along the z axis through the center of mass of the peptide terminus (Gromacs geometry option cylinder; see Fig. S1 for a schematic).

The initial configurations of umbrella sampling windows for each insertion were obtained by pulling a single peptide terminus through the membrane. The terminus was pulled for 1 μs with a pulling rate of 4.2 nm μs^{-1}. The initial reference distance for the harmonic potential was roughly $\pm$ 2.3 (depending on the membrane thickness), and its force constant was 5000 kJ mol^{-1} nm^{-2}.

For adsorption simulations, the initial configurations for umbrella sampling were obtained by pulling a peptide terminus away from the membrane for 500 ns with a pulling rate of 8.0 nm μs^{-1}. Initial reference distance for the harmonic potential was roughly ± 2.0 (depending on the membrane thickness), and its force constant was 5000 kJ mol^{-1} nm^{-2}.

Each insertion process was sampled using roughly 64 umbrella sampling windows (the exact number varied depending on the membrane thickness). The umbrella sampling windows were nonuniformly distributed along the range of the collective variable, with spacing of 0.1 nm near the membrane surface and close to the transmembrane state of the peptide and a spacing of 0.05 nm near the center of the membrane. Three different force constants were used: 1000 kJ mol^{-1} nm^{-2} for windows near the membrane surface and close to the transmembrane state, 5000 kJ mol^{-1} nm^{-2} for windows near the membrane center, and 3000 kJ mol^{-1} nm^{-2} for windows in intermediate areas. Each umbrella sampling window was simulated for 2 (most systems), 1 (systems with an asymmetric POPC membrane), 3 (systems with the LS peptide and asymmetric POPC/PUPC membrane), or 4 μs (systems with the LK peptide and large POPG and large asymmetric POPE/POPG membranes) with the first 10 ns being used for equilibration only. A full list of umbrella sampling windows with their reference positions and force constants used for insertion simulations is shown in Table S1.

Each adsorption process was sampled by roughly 32 umbrella sampling windows, nonuniformly distributed along the range of the collective variable. The spacing between the windows was 0.1 nm near the membrane surface and 0.2 nm in the solvent. Three different force constants were applied: 1000 kJ mol^{-1} nm^{-2} for windows near the membrane surface, 200 kJ mol^{-1} nm^{-2} in the solvent area, and 500 kJ mol^{-1} nm^{-2} in intermediate windows. Each umbrella sampling window was simulated for 2 (most systems), 1 (systems with asymmetric POPC membrane), 3 (systems with the LS peptide and asymmetric POPC/PUPC membrane), or 4 μs (systems with the LS peptide and POPE, POPG, or POPE/POPG membranes; systems with the LK peptide and large POPG and large asymmetric POPE/POPG membranes), with the first 10 ns being used for equilibration only. A full list of umbrella sampling windows with their reference positions and force constants used for adsorption simulations is shown in Table S2.

A free energy profile for each individual insertion/adsorption was obtained from a set of umbrella sampling windows using the weighted histogram analysis method (33,34) as implemented in the Grossfield Lab WHAM program (available from hembrane.urmc.rochester.edu). Full translocation profiles were obtained by aligning and joining the individual subprocesses using in-house-developed scripts. For a more detailed description of the aligning process, refer to Fig. S2.

For each peptide, we conducted two additional pulling simulations in the reverse direction to ensure the absence of hysteresis in the translocation process. In the first "backward" pull, the peptide began in a transmembrane state with its N-terminus in the lower leaflet. The N-terminus was then pulled upwards, forcing the peptide to exit the membrane and adopt the adsorbed state. In the second "backward" pull, the peptide started in solution, and its N-terminus was pulled toward the membrane. We used configurations from these trajectories as starting points for further umbrella sampling simulations. Comparing the free energies from the "forward" (standard) and "backward" pulls, we found no significant hysteresis, as depicted in Fig. S3.

Coarse-grained simulations of lipid flip-flop

The coarse-grained simulations of lipid flip-flop were performed similarly to the simulations of peptide translocation. We will thus only highlight the differences between these two.

We used three different membranes: symmetric POPC, asymmetric POPC/DGPC (both with the same composition as described for peptide translocation), and symmetric DGPC membrane with one DGPC lipid from the upper leaflet replaced by a single POPC lipid (containing 143 DGPC +1 POPC in the upper leaflet and 144 DGPC in the lower leaflet).

After membrane equilibration (described previously), a POPC lipid from the upper membrane leaflet was selected and pulled through the membrane for 1 μs. The collective variable was the oriented distance between the selected lipid phosphate and the local membrane center of mass on the z axis (radius of 2.0 nm). The initial reference distance for the harmonic potential was 2.3 (symmetric POPC) or 2.4 nm (symmetric DGPC or asymmetric POPC/DGPC), and its force constant was 5000 kJ mol^{-1} nm^{-2}. The pulling rate was 4.6 (symmetric POPC) or 4.8 nm μs^{-1} (symmetric DGPC or asymmetric POPC/DGPC).

The flip-flop process was sampled using 67 (symmetric POPC) or 69 (asymmetric DGPC or asymmetric POPC/DGPC) umbrella sampling windows. These were nonuniformly distributed along the range of the collective variable with spacing of 0.1 nm for windows near the membrane surface and 0.05 nm for windows near the membrane center. Force constants used ranged from 1000 kJ mol^{-1} nm^{-2} for windows near the membrane surface to 4000 kJ mol^{-1} nm^{-2} for windows near the membrane center. Each umbrella sampling window was simulated for 1 μs, with the first 10 ns being used for equilibration only. See Table S3 for a full list of umbrella sampling windows used.

Atomistic simulations of lipid flip-flop

For atomistic simulations, we employed the CHARMM36m force field (35). As with coarse-grained simulations, three membranes were used, all constructed using the CHARMM-GUI web interface (36). The symmetric POPC membrane was composed of 64 POPC lipids in each leaflet, the symmetric DGPC membrane consisted of 63 DGPC lipids and 1 POPC lipid in the upper leaflet and 64 DGPC lipids in the lower leaflet, and the asymmetric POPC/DGPC membrane contained 64 POPC lipids in the upper leaflet and 62 DGPC lipids in the lower leaflet. Each membrane system further contained roughly 5000 molecules of water and NaCl ions at a concentration of 0.154 mol dm^{-3}. The approximate size of each system was 6.5 $\times$ 6.5 $\times$ 8.0 nm.

The potential energy of each system was minimized using the steepest-descent algorithm with a force tolerance of 1000 kJ mol^{-1} nm^{-1}. During the minimization, position restraints were applied to the phosphorus atom

of each lipid (force constant of 1000 kJ mol^{-1} nm^{-2}). Dihedral restraints (force constant of 1000 kJ mol^{-1} rad^{-2}) were further applied to two dihedral angles in all POPC molecules and to three dihedrals in all DGPC molecules. The restrained POPC dihedrals were between C1, C3, C2, and O21 (glycerol carbons and oxygen linking the oleoyl tail to glycerol) and between C28, C29, C210, and C211 (carbons around the double bond of the oleyol tail). The C1-C3-C2-O21 dihedral was fixed at $-120^\circ \pm 2.5^\circ$, while the C28-C29-C210-C211 was fixed at $0^\circ \pm 0.0^\circ$. The restrained DGPC dihedrals were between C1, C3, C2, and O21 (the same as for POPC), between C210, C211, C212, and C213, and between C310, C311, C312, and C313 (carbons around the double bonds of the eicosenoyl tails). The C1-C3-C2-O21 dihedral was fixed at $-120^\circ \pm 2.5^\circ$, while the other two dihedrals were fixed at $0^\circ \pm 0.0^\circ$.

Equilibration was carried out in six stages of varying simulation lengths: stages 1–3 were each 250 ps long, while stages 4 and 5 were 1 ns long, and stage 6 was 5 ns long. Stages 1–2 were performed in the NVT ensemble, while stages 3–6 were performed in the NPT ensemble. A stochastic velocity rescaling thermostat (29) with a coupling constant of 0.5 ps was employed to maintain the temperature at 310 K. Two separate thermal baths were used for water with ions and membrane, respectively. During NPT stages of equilibration, the pressure was maintained at 1 bar using the Berendsen barostat (26) with semi-isotropic pressure coupling, a coupling constant of 5 ps, and a compressibility of 4.5×10^{-5} bar^{-1}. The equations of motion were integrated using the leap-frog algorithm. The simulation time step was 1 fs in stages 1–3, while it was 2 fs in the rest of the equilibration and following simulations. Short-ranged nonbonded interactions were truncated at 1.2 nm, and a force switch was applied starting from 1.0 nm. Long range electrostatic interactions were treated using fast smooth particle-mesh Ewald (37). Bonds with hydrogens were constrained using the LINCS algorithm (38). Translational velocity removal was applied separately for membrane and water with ions. Position and dihedral restraints were strong in the initial stages of the equilibration and were gradually reduced (position restraints: 1000 → 400 → 400 → 200 → 40 → 0, all in kJ mol^{-1} nm^{-2}; dihedral restraints: 1000 → 400 → 200 → 200 → 100 → 0, all in kJ mol^{-1} rad^{-2}).

Equilibration was followed by 100-ns-long molecular dynamics with production parameters. The Berendsen thermostat was replaced with the Parrinello-Rahman barostat (27,28), and no restraints were applied to the system. All other simulation settings remained the same as in stage 6 of equilibration.

As with coarse-grained simulations, the free energy of the flip-flop process was calculated using the umbrella sampling method (31,32). To enhance the sampling of the water defect formed during the lipid translocation (see Fig. S4 for simulation snapshots), we used the Plumed plugin v2.7.2 (39) and applied Hamiltonian replica exchange (40) to 16 windows near the membrane center (see Table S4). The initial configurations for the umbrella sampling windows were generated by pulling a selected POPC lipid from the upper membrane leaflet to the lower membrane leaflet. The collective variable was the oriented distance between the selected lipid phosphorus atom and the local membrane center of mass on z axis (radius of 2.0 nm). The pulling rate was 9.2 nm μs^{-1} with an initial reference distance of 2.3 nm and a force constant of 5000 kJ mol^{-1} nm^{-2}. The pulling was performed for 500 ns.

The pulling trajectory was split into 59 nonuniformly distributed umbrella sampling windows with spacing ranging from 0.1 nm near the membrane surface to 0.03 nm near the membrane center. The force constant of 1000 kJ mol^{-1} nm^{-2} was applied in windows near the membrane surface, while the force constant of 2000 kJ mol^{-1} nm^{-2} was used for windows near the membrane center. Hamiltonian replica exchange was applied to 16 windows in the membrane center (between -0.25 and 0.21 nm). The exchange of configurations was attempted every 100,000 integration steps (200 ps). Each umbrella sampling window was simulated for 300 (symmetric POPC, symmetric DGPC) or 400 ns (asymmetric POPC/DGPC), with the first 50 ns being used for equilibration only. See Table S4 for a full list of umbrella sampling windows used.

The free energy profiles were obtained in the same way as for coarse-grained simulations: using the weighted histogram analysis method (33,34) and the Grossfield Lab WHAM program (available from membrane.urmc.rochester.edu).

Pressure profile calculations

To further characterize the properties of symmetric and asymmetric membranes, we simulated each equilibrated Martini membrane (without peptide) for an additional 1 μs using Gromacs 2016.4. Subsequently, we calculated the stresses in each membrane employing Gromacs-LS (2016 version) (41). Lateral pressure, P_L, was determined as $P_L = -(\sigma_{xx} + \sigma_{yy})/2$, where σ represents the stress in the respective dimension. Normal pressure, P_N, was derived as $P_N = -\sigma_{zz}$.

The tension of the upper leaflet, γ_+, was calculated using the integral $\int_0^\infty (P_N - P_L)dz$, and the tension of the lower leaflet, γ_-, was determined with $\int_{-\infty}^0 (P_N - P_L)dz$, where $z = 0$ corresponds to the midplane of the membrane. The position of the membrane midplane was ascertained from the lipid tail densities along the normal of the membrane.

RESULTS AND DISCUSSION

Studied systems

Using coarse-grained Martini 3 force field (23), we calculated translocation free energy profiles across several symmetric and asymmetric membranes. We employed two α-helical peptides—LS and LK. LS is an amphiphilic peptide comprising leucine and serine residues (LSSLLSLLSS LLSLLSSLLSL-NH2), while LK consists of leucine and lysine residues (LKKLLKLLKKLLKLLKKLLKL-NH2) with a net charge of +10. Both peptides had a positively charged N-terminus and amidated (neutral) C-terminus. For simplicity, we used asymmetric membranes comprising two lipid types, with one leaflet entirely composed of one lipid type and the other leaflet entirely composed of another lipid type. The two-component asymmetric membranes were constructed with matching leaflet surface areas, leading to leaflet tensions smaller than ± 4 mN/m. Refer to Fig. S5 for the calculated pressure profiles and the specific values of leaflet tensions for each Martini membrane.

We supplemented the results by calculating the translocation free energy profiles across symmetric membranes each composed of one lipid type. In addition, we used a symmetrized/scrambled membrane made of lipid mixture of both lipid types. In total, we used five distinct lipid species: POPC, POPE, POPG, DGPC, and PUPC.

Translocation pathway

Both LS and LK peptides exhibited similar translocation pathways across all simulated membranes, which was previously described in peptide translocation studies across symmetric membranes (9,11,12,42,43). Fig. 1 *A* provides a schematic representation of the translocation process across the membranes. Initially, the peptides adsorb onto the membrane surface parallel to it, with their hydrophilic or charged

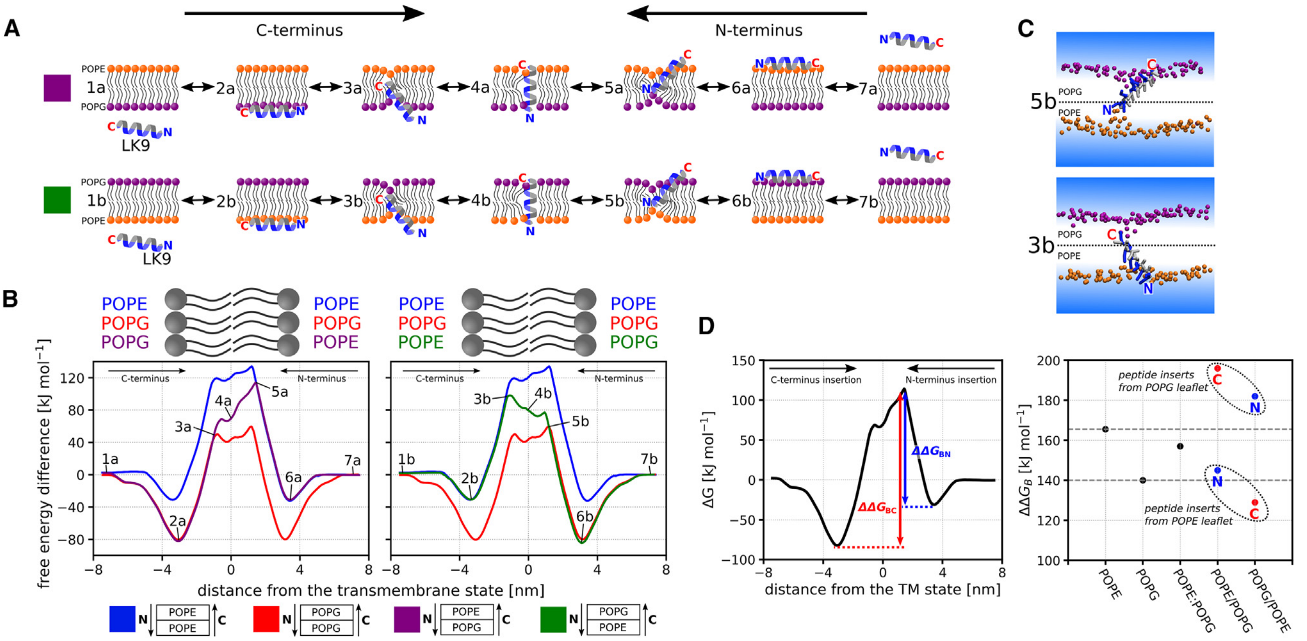

FIGURE 1 Translocation of the positively charged LK peptide across symmetric and asymmetric membranes composed of POPE and POPG lipids. (*A*) Schematic representation of the LK peptide translocating across an asymmetric membrane with POPE lipids in the upper leaflet and POPG lipids in the lower leaflet (POPE/POPG membrane) and across a membrane with the inverse composition (POPG/POPE membrane). The peptide is shown with its C-terminus entering the lower leaflet; however, it can also insert with its N-terminus first. In such cases, the process is equivalent to translocation across a membrane with inverse lipid composition, as the solvent environment on both sides of the simulated membrane is identical. (*B*) Free energy profiles of the LK peptide translocating across pure POPE (*blue*), pure POPG (*red*), asymmetric POPE/POPG (*purple*), and asymmetric POPG/POPE (*green*) membranes, with a calculation error below 5 kJ mol^{-1}. We show the profiles for the asymmetric POPE/POPG (*purple*) and for the asymmetric POPG/POPE (*green*) membranes in two separate charts for visual clarity. The labels correspond to specific translocation states depicted in (*A*). See Fig. S6 for the complete set of free energy profiles of LK translocation, including those for the symmetric POPE:POPG 1:1 membrane, Fig. S7 for convergence data and additional information, and Fig. S3 to verify the absence of hysteresis in our results. (*C*) Two simulation snapshots illustrating the LK peptide's position at points where its translocation free energy profile in the asymmetric membrane starts diverging from that in the symmetric membrane. Note how the peptide draws phosphates from the opposite membrane leaflet toward its inserting terminus. (*D*) Left: the translocation barrier, $\Delta\Delta G_B$, represents the highest difference between a local free energy maximum and minimum that the peptide must overcome to cross the membrane. In asymmetric membranes, there are two distinct translocation barriers, one for each translocation direction, denoted $\Delta\Delta G_{BN}$ and $\Delta\Delta G_{BC}$, based on the initially inserting peptide terminus. Right: translocation barriers, $\Delta\Delta G_B$, for the LK peptide in pure POPE, pure POPG, symmetric POPE:POPG 1:1, asymmetric POPE/POPG, and asymmetric POPG/POPE membranes. The gray horizontal dashed lines represent barriers for pure POPE and POPG membranes. Note that the translocation barriers for asymmetric membranes typically do not fall between those for the corresponding pure membranes; they can be substantially higher or lower, depending on which leaflet the peptide inserts into the membrane from. This suggests that for the LK peptide, the permeability of an asymmetric membrane cannot be deduced from the permeabilities of the pure membranes. For comprehensive details on translocation barriers and other relevant free energy values, see Fig. S20 and Table S5. To see this figure in color, go online.

residues facing the solvent (2a). Subsequently, one of the peptide termini inserts into the membrane (3a), accompanied by a change of the peptide orientation. In the transmembrane state (4a), the peptide is perpendicular to the membrane surface. The process of leaving the membrane mirrors the insertion process.

Characteristics of the translocation free energy profiles

Fig. 1 *B* shows the free energy profiles of the LK peptide translocating across POPE, POPG, and asymmetric POPE/POPG membranes (see also Fig. S6 for all free energy profiles calculated for the LK peptide, Fig. S7 for convergence of the calculations, and Fig. S3, which shows that no hysteresis is present in our results). The profile of translocation across asymmetric membranes with a small differential stress can be divided into three distinct regions: 1) a mimicking symmetric profile made of the upper leaflet, 2) a mimicking symmetric profile made of the lower leaflet, and 3) an intermediate region that is distinct from any symmetric membrane. In regions 1 and 2, the translocation process is indistinguishable from a symmetric, one-component membrane (further referred to as "pure membrane") comprising only the lipid type that constitutes the upper or lower leaflet of the asymmetric membrane, respectively. In other words, these regions of peptide translocation across the asymmetric membrane can be characterized well by determining the translocation across the corresponding symmetric membrane.

The peptide in these mimicking regions remains unaffected by the lipids of the opposite leaflet of the membrane,

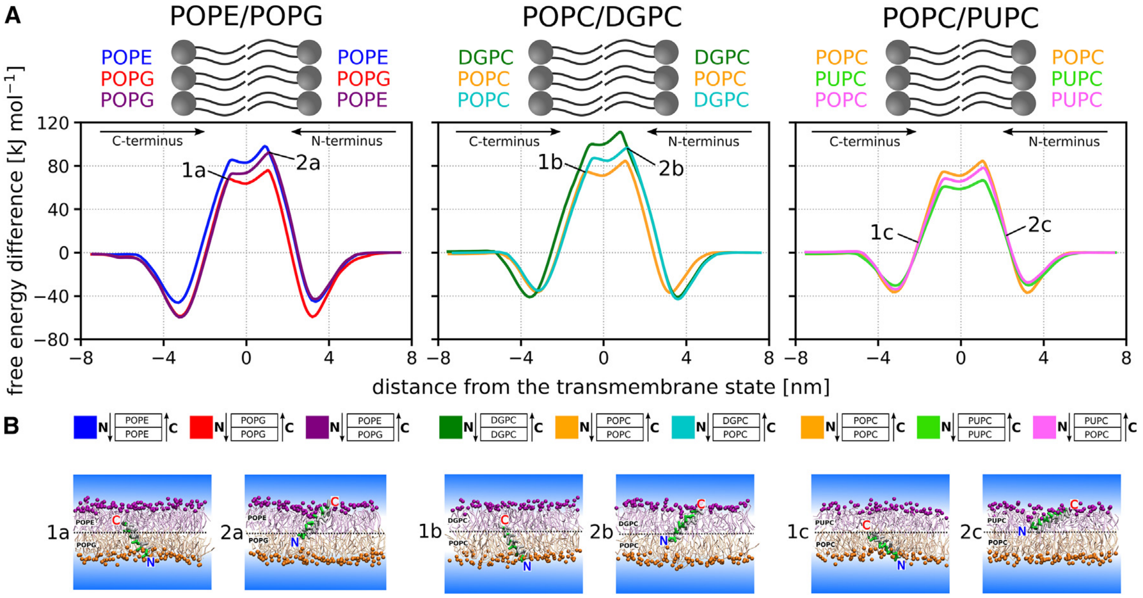

FIGURE 2 Translocation of the LS peptide, composed of leucines and serines, across various membranes. The left image shows translocation across POPE/POPG membranes composed of lipids with different headgroups but the same tails (POPE: C16:0/C18:1 PE vs. POPG: C16:0/C18:1 PG). The middle image shows translocation across POPC/DGPC membranes composed of lipids with different tail lengths and saturation in the central part of the tails (POPC: C16:0/C18:1 PC vs. DGPC: di-C20:1 PC). The right image shows translocation across POPC/PUPC membranes composed of lipids that differ along the entire tail segment in one of the acyl chains (POPC: C16:0/C18:1 PC vs. PUPC: C16:0/C22:6 PC). (*A*) Free energy profiles of the LS peptide translocating across the symmetric and asymmetric membranes mentioned above. The calculation error is below 5 kJ mol^{-1}. See also Figs. S13–S15 for all calculated free energy profiles of LS translocation, Figs. S16–S18 for the convergence of the calculations and additional details, and Fig. S3, which shows that there is no hysteresis in our results. (*B*) Simulation snapshots depicting the position of the LS peptide at translocation states where the free energy profile across the asymmetric membrane starts to deviate from the translocation profile across the corresponding symmetric membrane. Note that when translocating across the asymmetric POPC/PUPC membrane, the profile starts deviating much sooner than in the case of POPE/POPG and POPC/DGPC membranes, and the peptide is significantly less inserted. To see this figure in color, go online.

although it can still interact with them (see Figs. S8–S11). The existence of these regions indicates that for peptide translocation, the properties of the leaflets of the asymmetric membrane do not significantly impact each other. To further support this claim, we compared lipid order parameters of the asymmetric and pure membranes. As depicted in Fig. S12, the order parameters for lipids in both membranes are quite similar, differing slightly only in the tail region. It should also be noted that the mimicking regions exist despite the fact that the employed asymmetric membranes display nonzero, though small, differential stress (see Fig. S5), signifying that small leaflet tensions do not significantly influence the translocation free energies.

In the intermediate region, the peptide is located deep in the membrane and in direct contact with lipids in both upper and lower leaflet (see Fig. 1 *C*). This results in a free energy profile that deviates from those of pure membranes or symmetric two-component membrane (see Fig. S6 for the free energy profile of the LK peptide translocating across a symmetric POPE:POPG 1:1 membrane). Instead, the profile undergoes a gradual transition from conforming to the translocation profile of one pure membrane to conforming to the translocation profile of the other pure membrane. This transition is governed by the proportion of interactions between the peptide and the lipids in the lower and upper leaflets.

The location and size of this intermediate region depend on the specific membrane composition and the properties of the peptide. For example, in the case of the LS peptide translocating across an asymmetric POPC/PUPC membrane, the free energy profile deviates much sooner from the profile corresponding to pure membranes than in the case of translocation across POPE/POPG or POPC/DGPC membranes (see Fig. 2). Therefore, it is difficult to predict the exact position of the intermediate region in advance. However, it can be expected that for a single peptide, the intermediate region will start sooner and be wider if the lipids composing the two membrane leaflets differ in the character of their tails (such as POPC vs. PUPC) rather than in headgroups (such as POPE vs. POPG). See also Figs. S13–S15 for all calculated free energy profiles of the LS peptide translocating across POPE/POPG, POPC/DGPC, and POPC/PUPC membranes and Figs. S16–S18 for their convergence.

To verify that the observed behavior is not significantly influenced by the size of the periodic system, we also conducted simulations of the LK peptide translocating across larger (roughly three times more lipids) POPE/POPG membranes. As demonstrated in Fig. S19, we did not observe any

notable differences between the free energy profiles calculated in normal-sized and larger systems.

Translocation barriers and difficulty of translocation

The difficulty of translocation, indicated by the height of the translocation barriers, cannot be always reliably approximated by simply averaging the translocation barriers for two pure membranes or by using a symmetric two-component membrane. Consider, for instance, the LK peptide moving across an asymmetric POPE/POPG membrane. As shown in Fig. 1 *D*, and if the membrane leaflet into which the peptide inserts is made up of POPG lipids, the translocation barrier stands at either 182 or 196 kJ mol^{-1}, depending on whether the peptide inserts with its N-terminus or C-terminus first. In this case, the peptide is more likely to insert with its N-terminus first due to the lower translocation barrier. Nonetheless, either of the barriers starkly contrasts with translocation barriers calculated for pure POPE and POPG membranes, which are 166 and 140 kJ mol^{-1}, respectively (averaging to 153 kJ mol^{-1}). The barrier for a symmetric POPE:POPG 1:1 membrane also significantly deviates, registering at 157 kJ mol^{-1}. Conversely, if the membrane's outer leaflet is comprised of POPE, then the translocation process is easier due to the peptide's reduced stability when adsorbed on the POPE leaflet. Here, the barrier amounts to 145 kJ mol^{-1} when the N-terminus inserts first and to 129 kJ mol^{-1} for the more likely translocation pathway with the C-terminus being inserted first. Although these translocation barriers are closer to the "average" value derived from the pure membranes, such a similarity is merely coincidental. See Fig. S20 and Table S5 for more details concerning the translocation barriers and the free energy values of interest.

This finding contrasts with the conventional method used to study the permeation of molecules across asymmetric membranes. Typically, the permeabilities of two pure membranes are measured, and then the permeability of the asymmetric membrane is determined using the equation $1/P_{AB} = 1/P_A + 1/P_B$. Here, P_{AB} represents the permeability of the entire asymmetric membrane AB, while P_A and P_B denote the permeabilities of the individual leaflets from the pure AA and BB membranes, respectively (44,45). Permeability is directly proportional to $\exp(-\Delta\Delta G_B)$, where $\Delta\Delta G_B$ is the translocation barrier. Clearly, the above approach to estimating permeabilities is not suitable for the LK peptide since it fails to differentiate between distinct translocation directions exhibiting dramatically different translocation barriers.

The observed discrepancy between the simulation results and the standard theoretical model originates from the different stabilities of the peptide's adsorbed states, which codetermine the height of the translocation barriers (see Fig. 1D). If we know the free energy profiles of peptide translocation through pure membranes, then we can estimate the barrier for the translocation of this peptide through an asymmetric membrane. This estimate is obtained by averaging the free energy maxima from pure membrane profiles and then subtracting the free energy of the minimum, i.e., the adsorbed state. The subtracted adsorbed state is taken from the pure membrane, the composition of which corresponds to the leaflet from which the peptide inserts into the asymmetric membrane.

Consequently, if the stability of the adsorbed states does not dramatically differ between the pure membranes, then the translocation barrier for the asymmetric membrane can in fact be roughly estimated by averaging the translocation barriers observed for the pure membranes. This is evident in the case of the LS peptide in POPC/DGPC and POPC/PUPC membranes, as detailed in Table S5. However, the sum of inverse permeabilities is unreliable when the stability of the adsorbed states differs, as demonstrated on the studied charged peptide (LK) interacting with charge-asymmetric membranes (POPE/POPG). This scenario is likely very common because most of the cell-penetrating peptides are charged and the plasma membranes typically contain charged lipids predominantly in one leaflet.

Number asymmetry and large differential stress

As it has recently been suggested that cell membranes may be asymmetric not just in lipid composition but also in the number of lipids (7), we investigated the translocation of the LS peptide across a one-component asymmetric membrane, where the leaflets differed in the number of lipids. Specifically, we used a POPC membrane with 15% asymmetry, i.e., containing 144 lipids in one leaflet and 106 lipids in the other leaflet. As expected, this membrane exhibited dramatically higher differential stress than the other asymmetric membranes with leaflet tensions of roughly $\pm$ 21 mN/m (see Fig. S5). We found that, in this case, the free energy profiles of peptide translocation across the asymmetric membrane did not match any significant part of the translocation profile for the symmetric POPC membrane (see Fig. 3 for the results and Fig. S21 for the convergence of the calculations), i.e., the intermediate region spans the entire membrane. This result could be anticipated due to the fact that neither leaflet in the asymmetric POPC membrane corresponded to any leaflet in the symmetric POPC membrane, given that one leaflet of the asymmetric membrane was contracted while the other one was stretched.

We anticipate similar behavior in other asymmetric membranes with significant differential stress. Nonetheless, even in the presence of large differential stress, predicting the peptide translocation barrier from symmetric membranes is still unreliable. In fact, these predictions might be even more challenging for such membranes, as the differential stress further alters the free energies of peptide translocation.

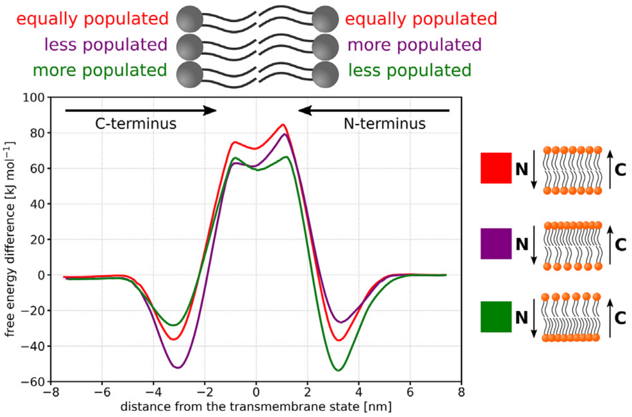

FIGURE 3 Free energy profiles of the LS peptide translocating through an symmetric POPC membrane (*red*) and through a POPC membrane with 15% number asymmetry (*purple* and *green*). Purple line corresponds to the peptide inserting its N-terminus into the more populated leaflet, and green line corresponds to the peptide inserting its N-terminus into the less populated leaflet. The asymmetric membrane displays large differential stress, which leads to the different free energy profiles within the entire membrane (including headgroups). See Fig. S21 for the free energy profiles of the individual translocation subprocesses and convergence of the calculations. To see this figure in color, go online.

Lipid flip-flop and wider implications

To explore whether the described translocation behavior extends to other molecules and to validate our results, we investigated phospholipid flip-flop in asymmetric and symmetric membranes. We employed both the coarse-grained Martini 3 (23) and atomistic CHARMM36m (35) force fields to compute the free energies of POPC flip-flop in pure POPC, pure DGPC, and asymmetric POPC/DGPC membranes. Similar to peptide translocation, we observed that the free energy profile of lipid flip-flop across asymmetric membrane initially conforms to the profiles for symmetric membranes and transitions between them in the center of the membrane (see Fig. 4 for the results and Fig. S22 for the convergence of the calculations). The width of the intermediate transition region was smaller for lipids than for peptides, likely due to the smaller size of the lipid molecules.

The existence of two mimicking regions and one intermediate region was observed with both coarse-grained and atomistic models, although the exact shape of the free energy profiles and height of the free energy barriers differed significantly between the models. These observations suggest that the presence of regions mimicking pure membranes and intermediate regions, as described for the translocating peptides in the Martini 3 force field, 1) are not artifacts of the coarse-grained model and 2) are applicable to other molecules that pass through the hydrophobic core of membranes.

Unlike the translocation barrier of the LK peptide in POPE/POPG membranes, but similar to the LS peptide in POPC/DGPC or POPC/PUPC membranes, we can approximate the flip-flop barrier for a POPC lipid in an asymmetric POPC/DGPC membrane using the barriers calculated in pure POPC and pure DGPC membranes. This approach is applicable due to the free energy minima being essentially equivalent on both sides of the asymmetric POPC/DGPC membrane. However, this may not be the case for all lipid types and membranes. If a lipid exhibits a significant

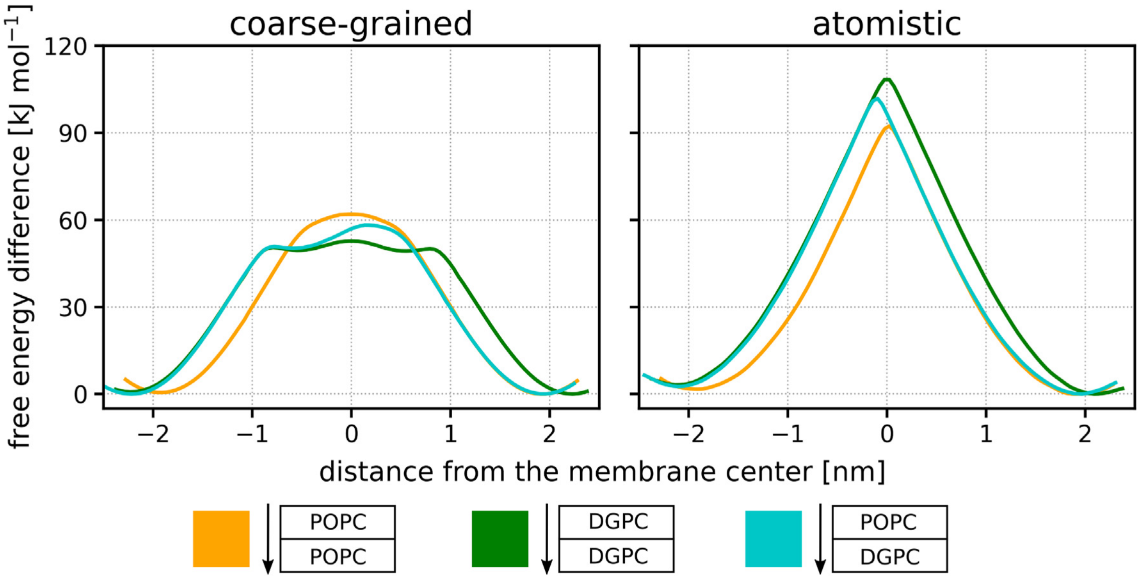

FIGURE 4 Free energy profiles of lipid flip-flop across symmetric POPC, symmetric DGPC, and asymmetric POPC/DGPC membranes calculated using the coarse-grained Martini 3 force field (*left*) and the atomistic CHARMM36m force field (*right*). The free energy of lipid flip-flop across asymmetric membrane follows the same trends as for the peptide translocation, with distinct regions mimicking pure membranes and the intermediate region. See Fig. S22 for the convergence of the calculations and Fig. S4 for simulation snapshots depicting flip-flop. To see this figure in color, go online.

preference for one leaflet, accurately approximating the flip-flop barrier for an asymmetric membrane using symmetric membranes becomes unreliable, similar to the LK and LS peptides in POPE/POPG membranes.

Although exploring the scrambling of other lipid types and molecules is beyond the scope of this study, we hypothesize that the permeation rate of any molecule significantly favoring one leaflet of an asymmetric membrane cannot be accurately estimated using permeation rates from symmetric membranes. Nonetheless, for many other molecules, it might be possible to reasonably approximate their permeation across asymmetric membranes as the sum of the inverse permeabilities of the symmetric membranes.

CONCLUSIONS

We employed the coarse-grained Martini 3 force field to calculate the translocation free energy of two model peptides across both symmetric and asymmetric membranes composed of lipids differing in their headgroups or acyl tails. In asymmetric membranes with a small differential stress, we identified regions of translocation that mimic translocation across symmetric membranes, as well as a region with unique behavior. Using both coarse-grained as well as atomistic free energy calculations, we show that phospholipids moving between the membrane leaflets exhibit similar behavior. For asymmetric membranes with a large differential stress, we observed that the leaflet tensions also significantly impact peptide adsorption, leading to distinct behavior throughout the entire asymmetric membrane. Our results also indicate that when a peptide adsorbs with varying strengths to the opposing leaflets of the asymmetric membrane, the translocation rate/barrier cannot be simply approximated using the translocation rates/barriers obtained for the corresponding symmetric membranes. Our findings provide insights into the behavior of molecules crossing asymmetric membranes and underscores the importance of considering membrane asymmetry when studying the permeation of biomolecules across cellular membranes.

SUPPORTING MATERIAL

Supporting material can be found online at https://doi.org/10.1016/j.bpj.2024.02.006.

AUTHOR CONTRIBUTIONS

L.B. carried out all the molecular dynamics simulations and analyzed the data. R.V. designed the research. L.B. and R.V. wrote the article.

ACKNOWLEDGMENTS

This work was supported by the European Research Council (ERC) under the European Union's Horizon 2020 research and innovation programme (grant agreement no. 101001470) and the project National Institute of Virology and Bacteriology (Programme EXCELES, ID project no. LX22NPO5103), funded by the European Union - Next Generation EU. Computational resources were provided by the CESNET, CERIT Scientific Cloud, and the IT4 Innovations National Supercomputing Center by MEYS CR through the e-INFRA CZ (ID: 90254). We acknowledge the use of the ChatGPT tool in rephrasing the article.

DECLARATION OF INTERESTS

The authors declare no competing interests.

REFERENCES

1. Wang, J., X. Dou, …, A. Shan. 2019. Antimicrobial peptides: Promising alternatives in the post feeding antibiotic era. *Med. Res. Rev.* 39:831–859. https://doi.org/10.1002/med.21542.
2. Bastos, P., F. Trindade, …, R. Vitorino. 2018. Human Antimicrobial Peptides in Bodily Fluids: Current Knowledge and Therapeutic Perspectives in the Postantibiotic Era. *Med. Res. Rev.* 38:101–146. https://doi.org/10.1002/med.21435.
3. Guidotti, G., L. Brambilla, and D. Rossi. 2017. Cell-Penetrating Peptides: From Basic Research to Clinics. *Trends Pharmacol. Sci.* 38:406–424. https://linkinghub.elsevier.com/retrieve/pii/S0165614717300172.
4. Tashima, T. 2017. Intelligent substance delivery into cells using cell-penetrating peptides. *Bioorg. Med. Chem. Lett.* 27:121–130. https://linkinghub.elsevier.com/retrieve/pii/S0960894X16312562.
5. Devaux, P. F. 1991. Static and dynamic lipid asymmetry in cell membranes. *Biochemistry.* 30:1163–1173. https://doi.org/10.1021/bi00219a001.
6. Lorent, J. H., K. R. Levental, …, I. Levental. 2020. Plasma membranes are asymmetric in lipid unsaturation, packing and protein shape. *Nat. Chem. Biol.* 16:644–652. http://www.nature.com/articles/s41589-020-0529-6.
7. Doktorova, M., J. L. Symons, …, I. Levental. 2023. Cell Membranes Sustain Phospholipid Imbalance Via Cholesterol Asymmetry. Preprint at bioRxiv. https://www.biorxiv.org/content/early/2023/07/31/2023.07.30.551157.
8. Brožek, R., I. Kabelka, and R. Vácha. 2020. Effect of Helical Kink on Peptide Translocation across Phospholipid Membranes. *J. Phys. Chem. B.* 124:5940–5947. https://doi.org/10.1021/acs.jpcb.0c03291.
9. Bartoš, L., I. Kabelka, and R. Vácha. 2021. Enhanced translocation of amphiphilic peptides across membranes by transmembrane proteins. *Biophys. J.* 120:2296–2305. https://www.sciencedirect.com/science/article/pii/S0006349521003003.
10. Cardenas, A. E., C. I. Drexler, …, R. Elber. 2022. Peptide Permeation across a Phosphocholine Membrane: An Atomically Detailed Mechanism Determined through Simulations and Supported by Experimentation. *J. Phys. Chem. B.* 126:2834–2849. https://doi.org/10.1021/acs.jpcb.1c10966.
11. Ulmschneider, J. P. 2017. Charged Antimicrobial Peptides Can Translocate across Membranes without Forming Channel-like Pores. *Biophys. J.* 113:73–81. https://linkinghub.elsevier.com/retrieve/pii/S0006349517306239.
12. Irudayam, S. J., and M. L. Berkowitz. 2012. Binding and reorientation of melittin in a POPC bilayer: Computer simulations. *Biochim. Biophys. Acta Biomembr.* 1818:2975–2981. https://linkinghub.elsevier.com/retrieve/pii/S0005273612002581.
13. Wheaten, S. A., F. D. O. Ablan, …, P. F. Almeida. 2013. Translocation of Cationic Amphipathic Peptides across the Membranes of Pure Phospholipid Giant Vesicles. *J. Am. Chem. Soc.* 135:16517–16525. https://doi.org/10.1021/ja407451c.

14. Thorén, P. E. G., D. Persson, …, B. Nordén. 2004. Membrane Binding and Translocation of Cell-Penetrating Peptides. *Biochemistry.* 43:3471–3489. https://doi.org/10.1021/bi0360049.

15. Marquardt, D., B. Geier, and G. Pabst. 2015. Asymmetric Lipid Membranes: Towards More Realistic Model Systems. *Membranes.* 5:180–196. http://www.mdpi.com/2077-0375/5/2/180.

16. Gurtovenko, A. A., and I. Vattulainen. 2007. Lipid Transmembrane Asymmetry and Intrinsic Membrane Potential: Two Sides of the Same Coin. *J. Am. Chem. Soc.* 129:5358–5359. https://doi.org/10.1021/ja070949m.

17. Vácha, R., M. L. Berkowitz, and P. Jungwirth. 2009. Molecular Model of a Cell Plasma Membrane With an Asymmetric Multicomponent Composition: Water Permeation and Ion Effects. *Biophys. J.* 96:4493–4501. https://linkinghub.elsevier.com/retrieve/pii/S0006349509007437.

18. Gurtovenko, A. A., and A. S. Lyulina. 2014. Electroporation of Asymmetric Phospholipid Membranes. *J. Phys. Chem. B.* 118:9909–9918. https://doi.org/10.1021/jp5028355.

19. Marrink, S. J., V. Corradi, …, M. S. P. Sansom. 2019. Computational Modeling of Realistic Cell Membranes. *Chem. Rev.* 119:6184–6226. https://doi.org/10.1021/acs.chemrev.8b00460.

20. Hossein, A., and M. Deserno. 2020. Spontaneous Curvature, Differential Stress, and Bending Modulus of Asymmetric Lipid Membranes. *Biophys. J.* 118:624–642. https://linkinghub.elsevier.com/retrieve/pii/S0006349519343929.

21. Pirhadi, E., J. M. Vanegas, …, X. Yong. 2023. Effect of Local Stress on Accurate Modeling of Bacterial Outer Membranes Using All-Atom Molecular Dynamics. *J. Chem. Theor. Comput.* 19:363–372. https://doi.org/10.1021/acs.jctc.2c01026.

22. Abraham, M. J., T. Murtola, …, E. Lindahl. 2015. GROMACS: High performance molecular simulations through multi-level parallelism from laptops to supercomputers. *SoftwareX.* 1–2:19–25. https://linkinghub.elsevier.com/retrieve/pii/S2352711015000059.

23. Souza, P. C. T., R. Alessandri, …, S. J. Marrink. 2021. Martini 3: a general purpose force field for coarse-grained molecular dynamics. *Nat. Methods.* 18:382–388. http://www.nature.com/articles/s41592-021-01098-3.

24. Pajtinka, P., and R. Vácha. 2024. Amphipathic Helices Can Sense Both Positive and Negative Curvatures of Lipid Membranes. *J. Phys. Chem. Lett.* 15:175–179. https://doi.org/10.1021/acs.jpclett.3c02785.

25. Šali, A., and T. L. Blundell. 1993. Comparative Protein Modelling by Satisfaction of Spatial Restraints. *J. Mol. Biol.* 234:779–815. https://doi.org/10.1006/jmbi.1993.1626.

26. Berendsen, H. J. C., J. P. M. Postma, …, J. R. Haak. 1984. Molecular dynamics with coupling to an external bath. *J. Chem. Phys.* 81:3684–3690. https://doi.org/10.1063/1.448118.

27. Parrinello, M., and A. Rahman. 1980. Crystal Structure and Pair Potentials: A Molecular-Dynamics Study. *Phys. Rev. Lett.* 45:1196–1199. https://doi.org/10.1103/physrevlett.45.1196.

28. Parrinello, M., and A. Rahman. 1981. Polymorphic transitions in single crystals: A new molecular dynamics method. *J. Appl. Phys.* 52:7182–7190. https://doi.org/10.1063/1.328693.

29. Bussi, G., D. Donadio, and M. Parrinello. 2007. Canonical sampling through velocity rescaling. *J. Chem. Phys.* 126, 014101. https://doi.org/10.1063/1.2408420.

30. Kim, H., B. Fábián, and G. Hummer. 2023. Neighbor List Artifacts in Molecular Dynamics Simulations. *J. Chem. Theor. Comput.* 19:8919–8929. https://doi.org/10.1021/acs.jctc.3c00777.

31. Torrie, G. M., and J. P. Valleau. 1974. Monte Carlo free energy estimates using non-Boltzmann sampling: Application to the sub-critical Lennard-Jones fluid. *Chem. Phys. Lett.* 28:578–581.

32. Torrie, G., and J. Valleau. 1977. Nonphysical sampling distributions in Monte Carlo free-energy estimation: Umbrella sampling. *J. Comput. Phys.* 23:187–199. https://linkinghub.elsevier.com/retrieve/pii/0021999177901218.

33. Kumar, S., J. M. Rosenberg, …, P. A. Kollman. 1992. The weighted histogram analysis method for free-energy calculations on biomolecules. I. The method. *J. Comput. Chem.* 13:1011–1021. https://doi.org/10.1002/jcc.540130812.

34. Souaille, M., and B. Roux. 2001. Extension to the weighted histogram analysis method: combining umbrella sampling with free energy calculations. *Comput. Phys. Commun.* 135:40–57. https://linkinghub.elsevier.com/retrieve/pii/S0010465500002150.

35. Huang, J., S. Rauscher, …, A. D. MacKerell. 2017. CHARMM36m: an improved force field for folded and intrinsically disordered proteins. *Nat. Methods.* 14:71–73. http://www.nature.com/articles/nmeth.4067.

36. Jo, S., T. Kim, …, W. Im. 2008. CHARMM-GUI: A web-based graphical user interface for CHARMM. *J. Comput. Chem.* 29:1859–1865. https://doi.org/10.1002/jcc.20945.

37. Essmann, U., L. Perera, …, L. G. Pedersen. 1995. A smooth particle mesh Ewald method. *J. Chem. Phys.* 103:8577–8593. https://pubs.aip.org/aip/jcp/article/103/19/8577-8593/180219.

38. Hess, B., H. Bekker, …, J. G. E. M. Fraaije. 1997. LINCS: A linear constraint solver for molecular simulations. *J. Comput. Chem.* 18:1463–1472. https://doi.org/10.1002/(SICI)1096-987X(199709)18:12<1463::AID-JCC4>3.0.CO;2-H.

39. Tribello, G. A., M. Bonomi, …, G. Bussi. 2014. PLUMED 2: New feathers for an old bird. *Comput. Phys. Commun.* 185:604–613. https://doi.org/10.1016/j.cpc.2013.09.018.

40. Fukunishi, H., O. Watanabe, and S. Takada. 2002. On the Hamiltonian replica exchange method for efficient sampling of biomolecular systems: Application to protein structure prediction. *J. Chem. Phys.* 116:9058–9067. https://doi.org/10.1063/1.1472510.

41. Vanegas, J. M., A. Torres-Sánchez, and M. Arroyo. 2014. Importance of Force Decomposition for Local Stress Calculations in Biomembrane Molecular Simulations. *J. Chem. Theor. Comput.* 10:691–702. https://doi.org/10.1021/ct4008926.

42. Kabelka, I., and R. Vácha. 2018. Optimal Hydrophobicity and Reorientation of Amphiphilic Peptides Translocating through Membrane. *Biophys. J.* 115:1045–1054. https://doi.org/10.1016/j.bpj.2018.08.012.

43. Chetwynd, A., C. L. Wee, …, M. S. P. Sansom. 2010. The Energetics of Transmembrane Helix Insertion into a Lipid Bilayer. *Biophys. J.* 99:2534–2540. https://linkinghub.elsevier.com/retrieve/pii/S0006349510009689.

44. Negrete, H. O., R. L. Rivers, …, M. L. Zeidel. 1996. Individual Leaflets of a Membrane Bilayer Can Independently Regulate Permeability. *J. Biol. Chem.* 271:11627–11630. https://linkinghub.elsevier.com/retrieve/pii/S0021925818825960.

45. Krylov, A. V., P. Pohl, …, W. G. Hill. 2001. Water Permeability of Asymmetric Planar Lipid Bilayers: Leaflets of Different Composition Offer Independent and Additive Resistances to Permeation. *J. Gen. Physiol.* 118:333–340.

Biophysical Journal, Volume *123*

Supplemental information

Peptide translocation across asymmetric phospholipid membranes

Ladislav Bartoš and Robert Vácha

SUPPORTING MATERIAL

Peptide translocation across asymmetric phospholipid membranes

Ladislav Bartoš and Robert Vácha

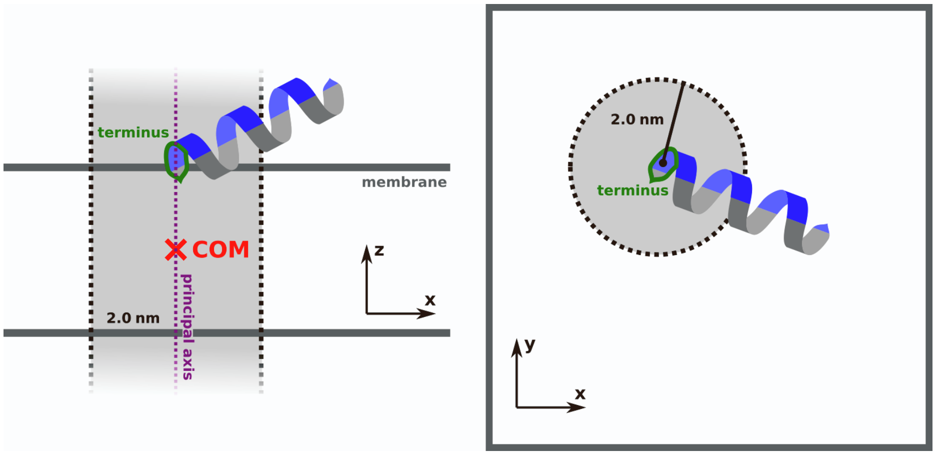

Figure S1: Schematic representation of the local membrane center of mass calculation. Left. Schematic side view of the membrane with peptide. Right. Schematic top view of the membrane with peptide. The local membrane center of mass (highlighted as red × symbol) is calculated from the positions of lipid beads localized inside a cylinder (highlighted in gray) with radius of 2.0 nm and its principal axis (purple dotted line) going along the z-axis through the center of mass of the inserting peptide terminus. Peptide terminus is defined as the first (N-terminus) or last (C-terminus) three backbone beads of the peptide and is highlighted in green. Note that the height of the cylinder (its dimension along the z-axis) is infinite.

Table S1: Distribution of umbrella sampling windows along the collective variable with biasing force constants used for insertion simulations of peptide translocation. Reference distances are in nm, force constants in kJ mol^{-1} nm^{-2}.

Reference distance	Force constant	Reference distance	Force constant
±2.50	1000*	±0.30	5000
±2.40	1000†	±0.25	5000
±2.30	1000#	±0.20	5000
±2.20	1000#	±0.15	5000
±2.10	1000#	±0.10	5000
±2.00	1000	±0.05	5000
±1.90	1000	0.00	5000
±1.80	1000	∓0.05	5000
±1.70	1000	∓0.10	5000
±1.60	1000	∓0.15	5000
±1.50	1000	∓0.20	5000
±1.45	3000	∓0.25	5000
±1.40	3000	∓0.30	5000
±1.35	3000	∓0.35	5000
±1.30	3000	∓0.40	5000
±1.25	3000	∓0.45	5000
±1.20	3000	∓0.50	3000
±1.15	3000	∓0.55	3000
±1.10	3000	∓0.60	3000
±1.05	3000	∓0.65	3000
±1.00	3000	∓0.70	3000
±0.95	3000	∓0.75	3000
±0.90	3000	∓0.80	3000
±0.85	3000	∓0.85	3000
±0.80	3000	∓0.90	3000
±0.75	3000	∓0.95	3000
±0.70	3000	∓1.00	1000
±0.65	3000	∓1.10	1000
±0.60	3000	∓1.20	1000
±0.55	3000	∓1.30	1000
±0.50	3000	∓1.40	1000
±0.45	5000	∓1.50	1000
±0.40	5000	∓1.60	1000*
±0.35	5000	∓1.70	1000*

* only used for symmetric DGPC, † only used for symmetric DGPC and asymmetric POPC/DGPC, # *not* used for symmetric PUPC

Table S2: Distribution of umbrella sampling windows along the collective variable with biasing force constants used for the adsorption simulations of peptide translocation. Reference distances are in nm, force constants in kJ mol^{-1} nm^{-2}.

Reference distance	Force constant	Reference distance	Force constant
±1.70	1000[#]	±3.50	500
±1.80	1000[#]	±3.60	500
±1.90	1000[#]	±3.70	500
±2.00	1000[*†]	±3.80	500
±2.10	1000[*]	±3.90	500
±2.20	1000	±4.00	500
±2.30	1000	±4.10	500
±2.40	1000	±4.20	200
±2.50	1000	±4.40	200
±2.60	500	±4.60	200
±2.70	500	±4.80	200
±2.80	500	±5.00	200
±2.90	500	±5.20	200
±3.00	500	±5.40	200
±3.10	500	±5.60	200
±3.20	500	±5.80	200
±3.30	500	±6.00	200
±3.40	500		

[#] only used for symmetric PUPC, [*] *not* used for symmetric DGPC, [†] *not* used for asymmetric POPC/DGPC

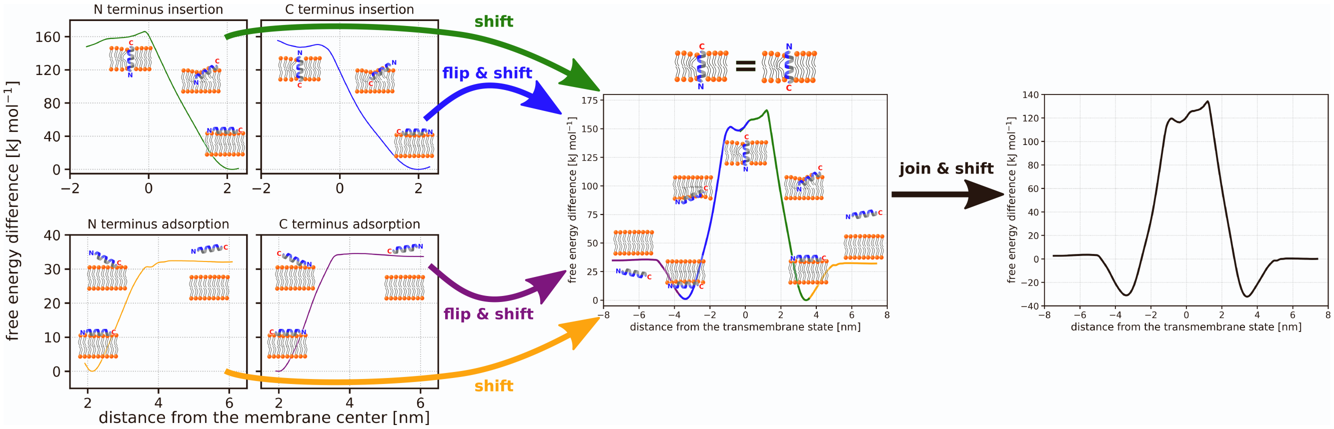

Figure S2: Example of the translocation profile construction demonstrated on free energy profiles calculated for the LK peptide translocating across symmetric POPE membrane. In symmetric membranes, the translocation process can be divided into four individual subprocesses: N-terminus insertion, C-terminus insertion, N-terminus adsorption, and C-terminus adsorption. To construct the complete translocation profile, we initially shift the profiles for N-terminus insertion and N-terminus adsorption in the x-dimension so that the transmembrane state of the peptide corresponds to $x = 0$. These aligned profiles are then joined with those for C-terminus insertion and adsorption. Since both N- and C-terminus insertion/adsorption are calculated using the peptide adsorbed on the same membrane leaflet, specifically the upper leaflet, the profiles for C-terminus insertion and adsorption must be flipped. This flipping is possible because the membrane is symmetric, and the leaflets are interchangeable. Thus, we flip the profiles for C-terminus insertion and adsorption, changing positive distance values to negative and vice versa. These flipped profiles represent insertion/adsorption from/to the lower leaflet. These profiles are again shifted in the x-dimension so that the transmembrane state is at $x = 0$. As illustrated in the middle chart, the free energy profiles of the individual subprocesses align almost perfectly, indicating only a small calculation error. We then combine the profiles and shift the ΔG values so that the free energy difference in the solvent (specifically in the solvent region of the N-terminus adsorption profile) equals zero. The free energy differences for peptide in solvent and in the adsorbed states on the opposite sides of the translocation profile provide a robust estimate of the error margin. It is important to note that in case of asymmetric membranes, the free energy profiles of subprocesses cannot be flipped. For asymmetric membranes, insertion/adsorption on both membrane leaflets is calculated, and appropriate free energy profiles are joined. The joining procedure for asymmetric membranes is otherwise equivalent to that described here.

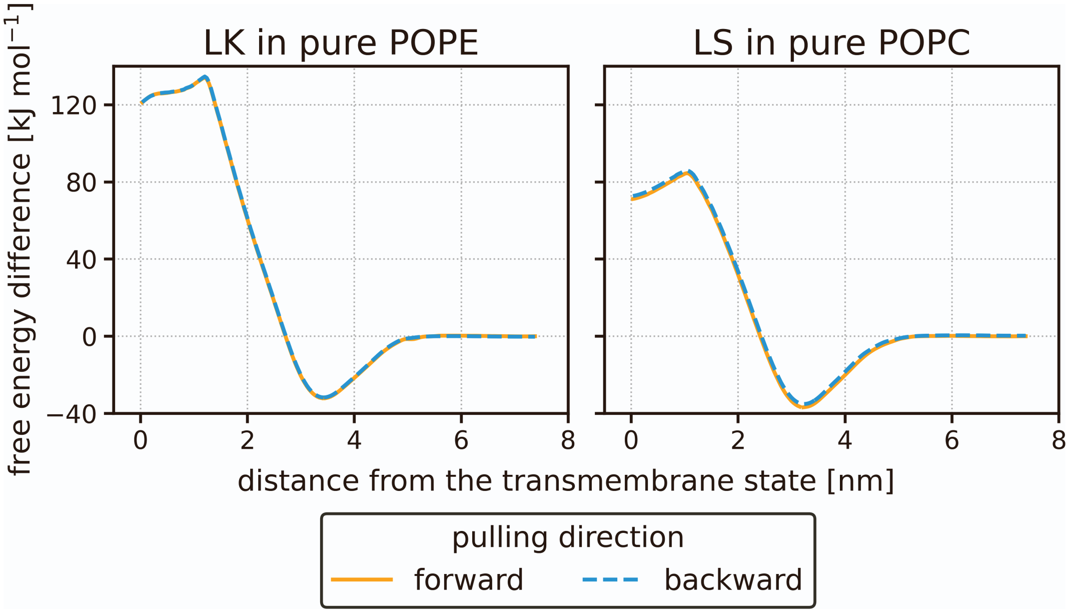

Figure S3: Free energy profiles of N-terminus insertion and adsorption of the LK peptide in a pure POPE membrane (left) and the LS peptide in a pure POPC membrane (right). The orange profiles were calculated from umbrella sampling windows which initial configurations were obtained from the standard "forward" pulling simulations. The blue dashed profiles were calculated from umbrella sampling windows with initial configurations from "backward" pulling simulations, performed in the opposite direction. For the LK and LS peptides, the maximum observed hysteresis was less than 1 kJ mol^{-1} and less than 2 kJ mol^{-1}, respectively. These values are significantly lower than our reported maximum error margin of 5 kJ mol^{-1}.

Table S3: Distribution of umbrella sampling windows along the collective variable with biasing force constants used for coarse-grained simulations of lipid flip-flop. Reference distances are in nm, force constants in kJ mol^{-1} nm^{-2}.

Reference distance	Force constant	Reference distance	Force constant
2.40	1000*	−0.05	4000
2.30	1000	−0.10	4000
2.20	1000	−0.15	3000
2.10	1000	−0.20	3000
2.00	1000	−0.25	3000
1.90	1000	−0.30	3000
1.80	1000	−0.35	2000
1.70	1000	−0.40	2000
1.60	1000	−0.45	2000
1.50	1000	−0.50	2000
1.40	1000	−0.55	2000
1.30	1000	−0.60	2000
1.20	1000	−0.65	2000
1.10	1000	−0.70	2000
1.00	2000	−0.75	2000
0.95	2000	−0.80	2000
0.90	2000	−0.85	2000
0.85	2000	−0.90	2000
0.80	2000	−0.95	2000
0.75	2000	−1.00	2000
0.70	2000	−1.10	1000
0.65	2000	−1.20	1000
0.60	2000	−1.30	1000
0.55	2000	−1.40	1000
0.50	2000	−1.50	1000
0.45	2000	−1.60	1000
0.40	2000	−1.70	1000
0.35	2000	−1.80	1000
0.30	2000	−1.90	1000
0.25	3000	−2.00	1000
0.20	3000	−2.10	1000
0.15	3000	−2.20	1000
0.10	4000	−2.30	1000
0.05	4000	−2.40	1000*
0.00	4000		

* *not* used for symmetric POPC

Table S4: Distribution of umbrella sampling windows along the collective variable with biasing force constants used for atomistic simulations of lipid flip-flop. Reference distances are in nm, force constants in kJ mol^{-1} nm^{-2}.

Reference distance	Force constant	Reference distance	Force constant
2.30	1000	−0.03	2000^R
2.20	1000	−0.06	2000^R
2.10	1000	−0.09	2000^R
2.00	1000	−0.12	2000^R
1.90	1000	−0.15	2000^R
1.80	1000	−0.18	2000^R
1.70	1000	−0.21	2000^R
1.60	1000	−0.25	2000^R
1.50	1000	−0.30	1000
1.40	1000	−0.40	1000
1.30	1000	−0.50	1000
1.20	1000	−0.60	1000
1.10	1000	−0.70	1000
1.00	1000	−0.80	1000
0.90	1000	−0.90	1000
0.80	1000	−1.00	1000
0.70	1000	−1.10	1000
0.60	1000	−1.20	1000
0.50	1000	−1.30	1000
0.40	1000	−1.40	1000
0.30	1000	−1.50	1000
0.25	2000	−1.60	1000
0.21	2000^R	−1.70	1000
0.18	2000^R	−1.80	1000
0.15	2000^R	−1.90	1000
0.12	2000^R	−2.00	1000
0.09	2000^R	−2.10	1000
0.06	2000^R	−2.20	1000
0.03	2000^R	−2.30	1000
0.00	2000^R		

^{R} windows where Hamiltonian replica exchange was applied

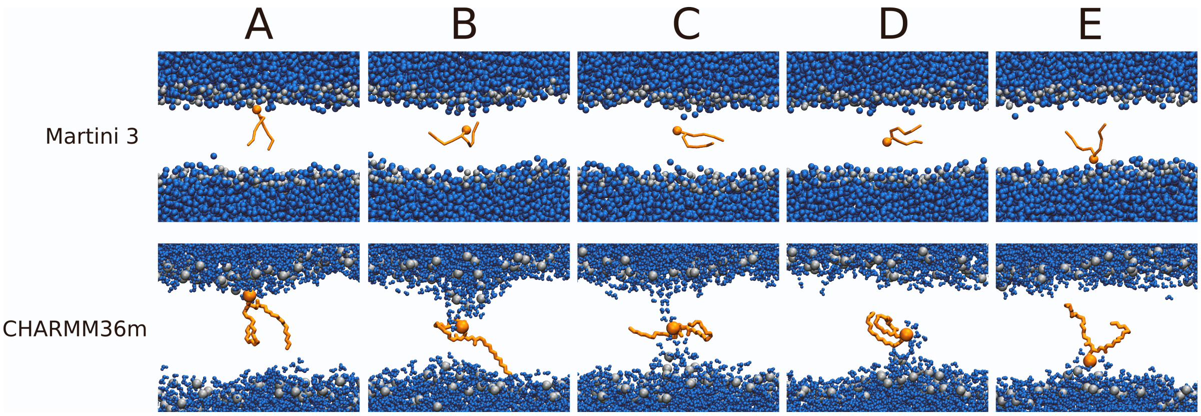

Figure S4: Representative simulation snapshots showing a POPC lipid translocating across a pure POPC membrane, taken from umbrella sampling windows employing the Martini 3 force field (upper row) or CHARMM36m force field (lower row). The snapshots are centered on the headgroup of the translocating lipid. This lipid is highlighted in orange, while only headgroups are shown for other lipids (gray beads). Water molecules are represented by blue beads. A particular emphasis is placed on configurations where the lipid is near the membrane center (panels B–D). In the Martini 3 simulations, there are no significant water defects, membrane disruptions, or water channels, but in the atomistic force field, large water defects are observed. The presence of the lipid at the membrane's center can lead to the formation of water defects from either the upper or the lower leaflet, or from both simultaneously, creating a water channel. The application of Hamiltonian replica exchange to atomistic umbrella sampling windows, where the lipid is near the membrane center, enhances the sampling of these distinct disrupted membrane structures.

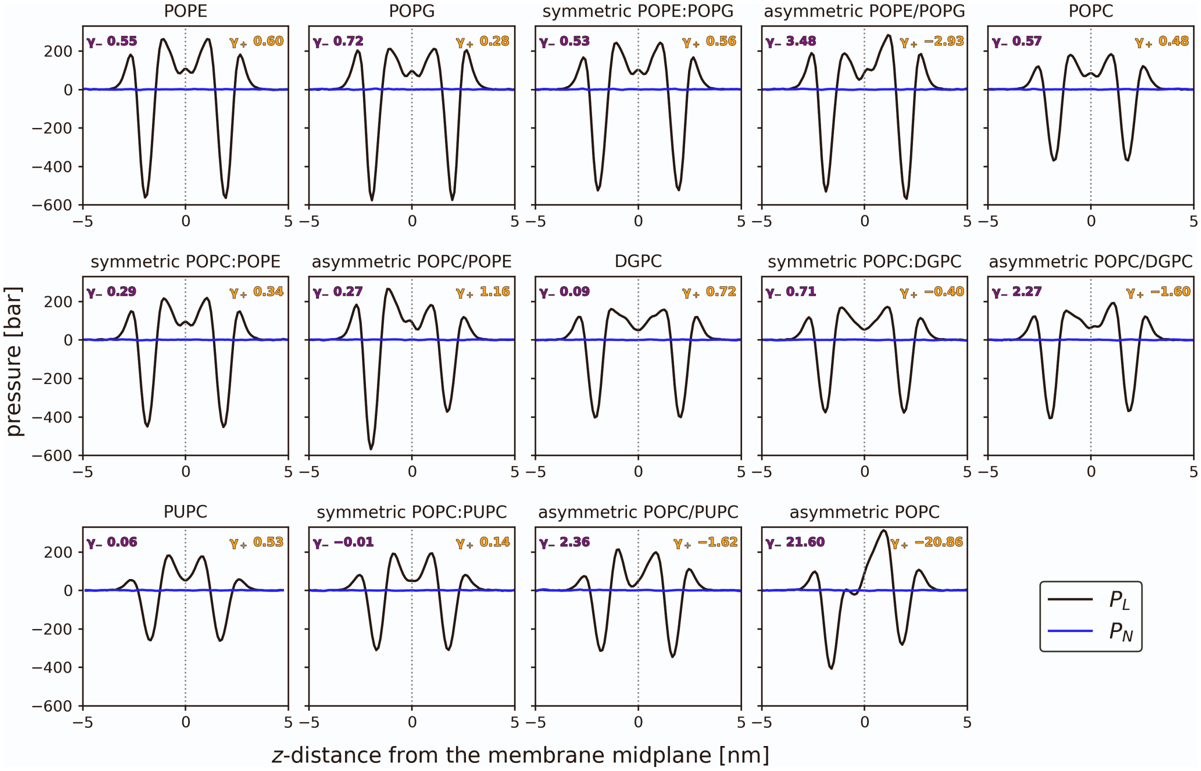

Figure S5: Lateral (P_L) and normal (P_N) pressure profiles along the membrane normal calculated for each peptide-free Martini membrane. Tension of the upper (γ_+, orange) and lower (γ_-, purple) leaflets are presented in units of mN/m. For details concerning the calculation procedure, refer to the Methods section. The calculation error was estimated from the symmetric membranes to be below 1 mN/m.

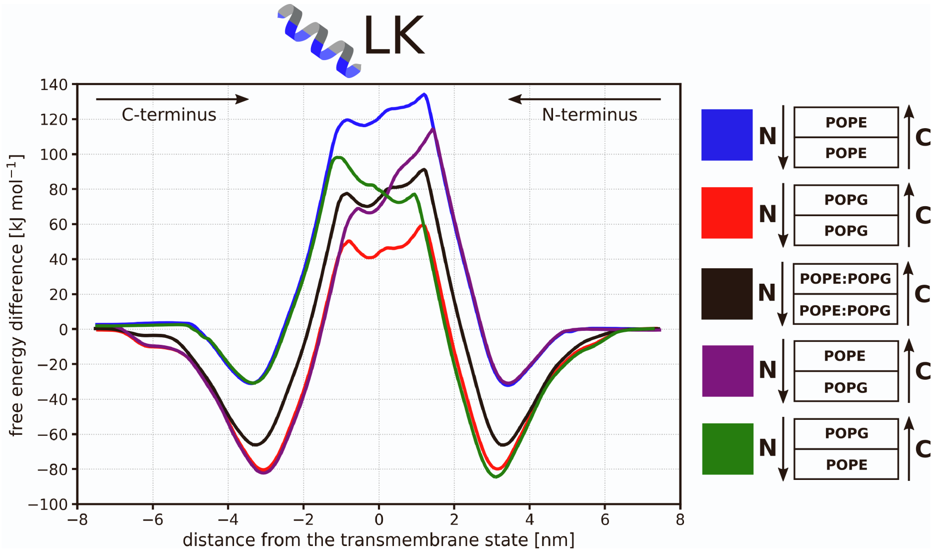

Figure S6: Free energy profiles of the LK peptide translocating through symmetric and asymmetric POPE/POPG membranes (blue = pure POPE, red = pure POPG, black = symmetric POPE:POPG 1:1, purple = asymmetric POPE/POPG, green = asymmetric POPG/POPE). The calculation error is below 5 kJ mol^{-1}. See Figure S7 for the free energy profiles of the individual translocation subprocesses and convergence of the calculations.

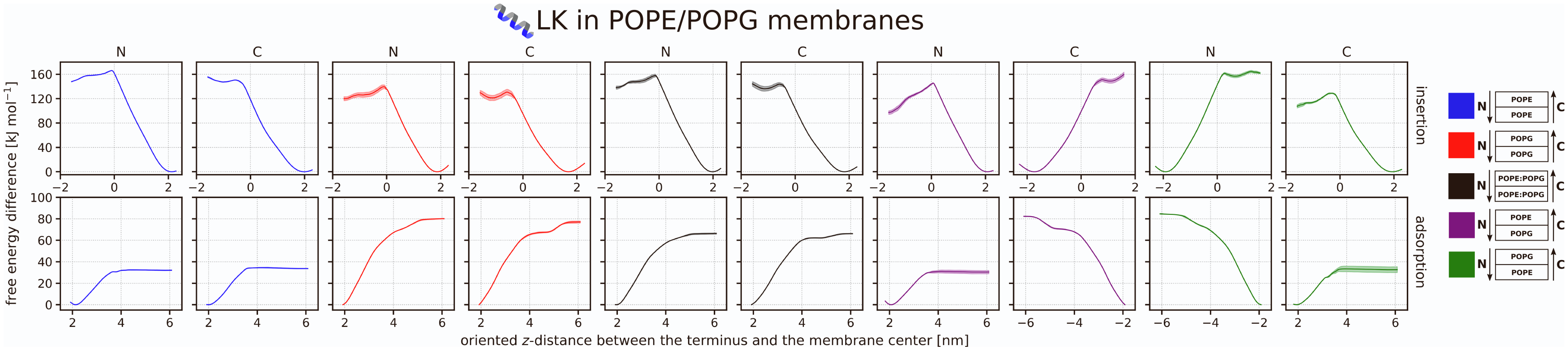

Figure S7: Free energy profiles for individual subprocesses of the LK peptide translocating through both symmetric and asymmetric POPE/POPG membranes. The full translocation profile is constructed by aligning and joining the individual subprocesses calculated for the same system, sharing the color. The color scheme is consistent with Figure S6. The first row presents the free energy profiles of peptide insertion, whereas the second row shows the free energy profiles of peptide adsorption. The columns labeled "N" and "C" correspond to the insertion/adsorption of the peptide's N-terminus and C-terminus, respectively. Shading depicts the convergence of the calculation as it corresponds to the difference between the free energy profiles calculated from the initial half and the latter half of the production phase of the umbrella sampling simulation.

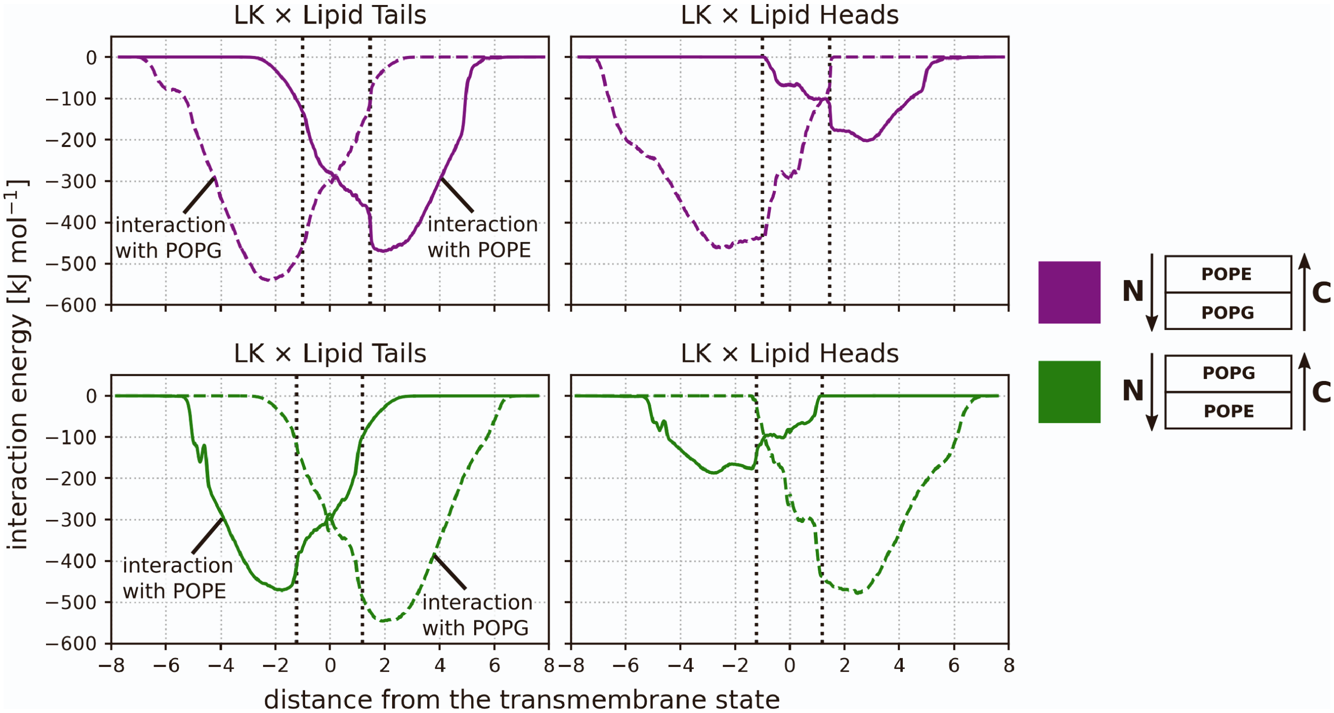

Figure S8: Interaction energy of the LK peptide translocating through asymmetric POPE/POPG membranes with phospholipid tails (left column) and heads (right column). The interaction energies were calculated from all umbrella sampling windows. Purple lines correspond to system in which the peptide translocated through POPE/POPG membrane, while green lines correspond to system in which the peptide translocated through POPG/POPE membrane. Full lines correspond to interaction with POPE lipids while dashed lines correspond to interaction with POPG lipids. Vertical dotted lines show approximate positions where the translocation profile for the asymmetric membrane differs from that for the symmetric membrane.

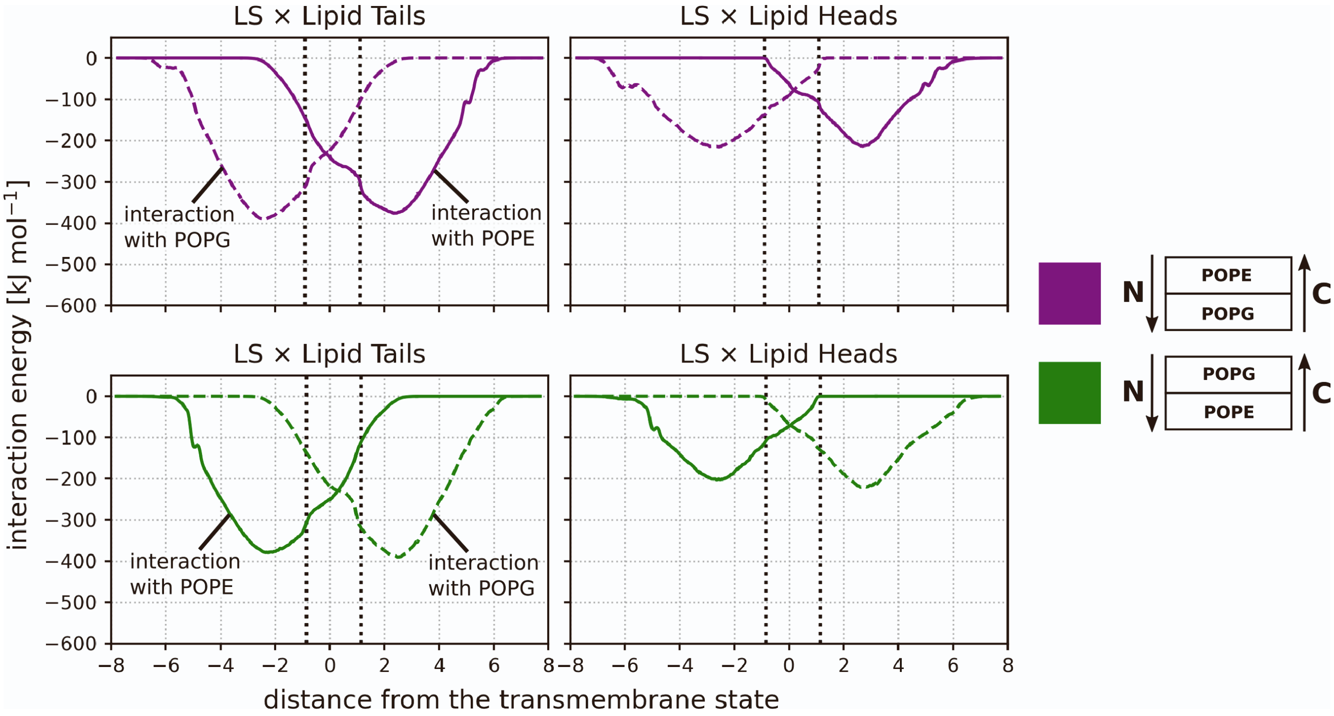

Figure S9: Interaction energy of the LS peptide translocating through asymmetric POPE/POPG membranes with phospholipid tails (left column) and heads (right column). The interaction energies were calculated from all umbrella sampling windows. Purple lines correspond to system in which the peptide translocated through POPE/POPG membrane, while green lines correspond to system in which the peptide translocated through POPG/POPE membrane. Full lines correspond to interaction with POPE lipids while dashed lines correspond to interaction with POPG lipids. Vertical dotted lines show approximate positions where the translocation profile for the asymmetric membrane differs from that for the symmetric membrane.

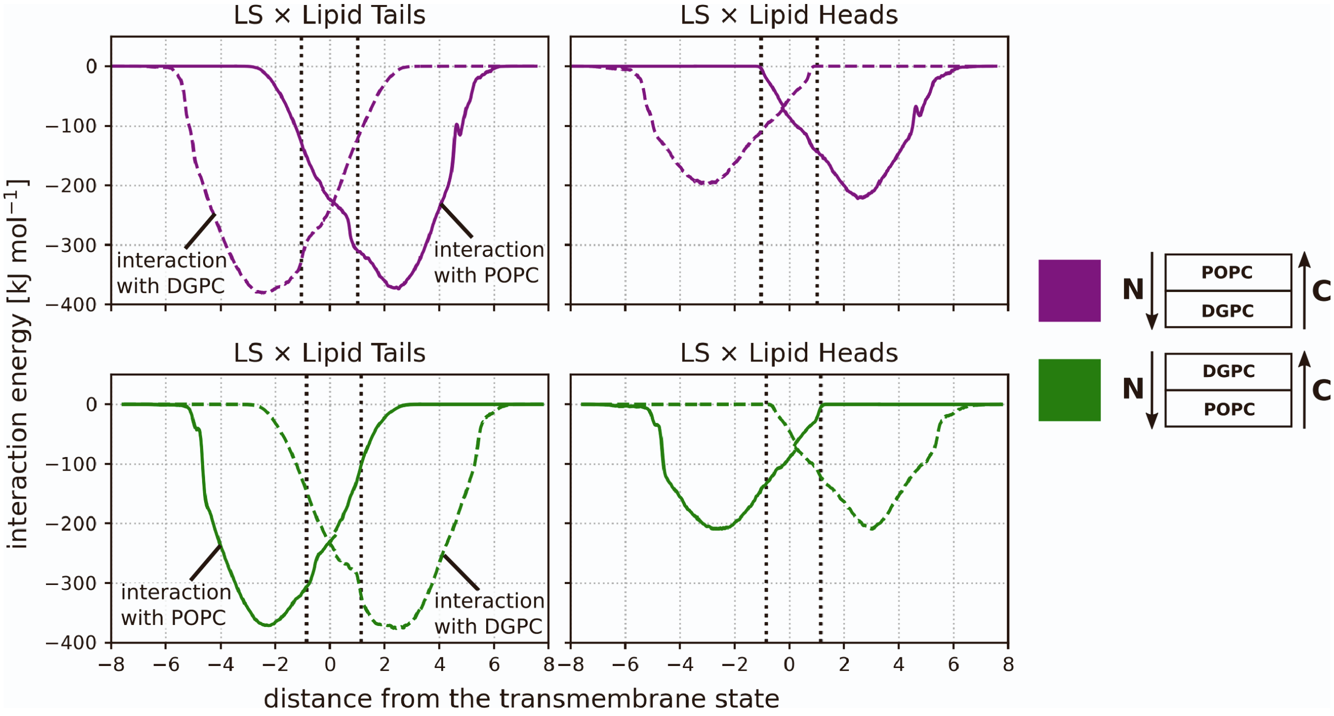

Figure S10: Interaction energy of the LS peptide translocating through asymmetric POPC/DGPC membranes with phospholipid tails (left column) and heads (right column). The interaction energies were calculated from all umbrella sampling windows. Purple lines correspond to system in which the peptide translocated through POPC/DGPC membrane, while green lines correspond to system in which the peptide translocated through DGPC/POPC membrane. Full lines correspond to interaction with POPC lipids while dashed lines correspond to interaction with DGPC lipids. Vertical dotted lines show approximate positions where the translocation profile for the asymmetric membrane differs from that for the symmetric membrane.

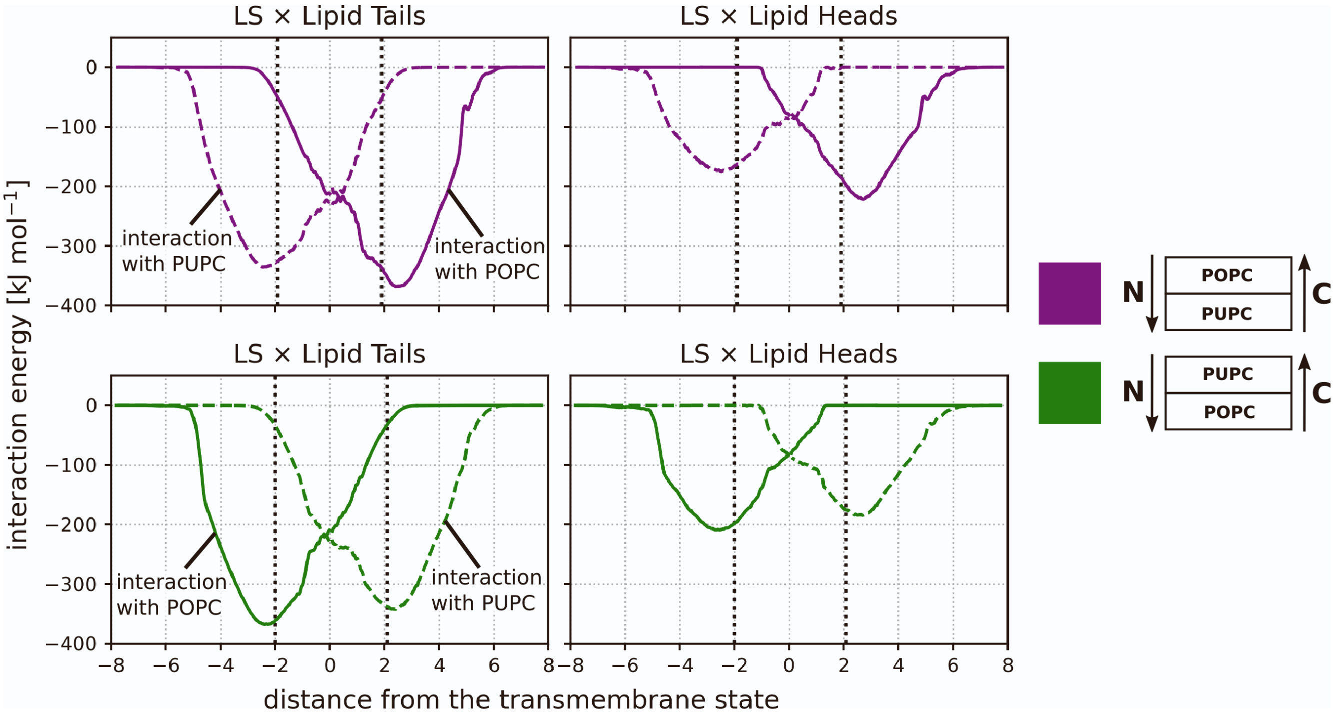

Figure S11: Interaction energy of the LS peptide translocating through asymmetric POPC/PUPC membranes with phospholipid tails (left column) and heads (right column). The interaction energies were calculated from all umbrella sampling windows. Purple lines correspond to system in which the peptide translocated through POPC/PUPC membrane, while green lines correspond to system in which the peptide translocated through PUPC/POPC membrane. Full lines correspond to interaction with POPC lipids while dashed lines correspond to interaction with PUPC lipids. Vertical dotted lines show approximate positions where the translocation profile for the asymmetric membrane differs from that for the symmetric membrane.

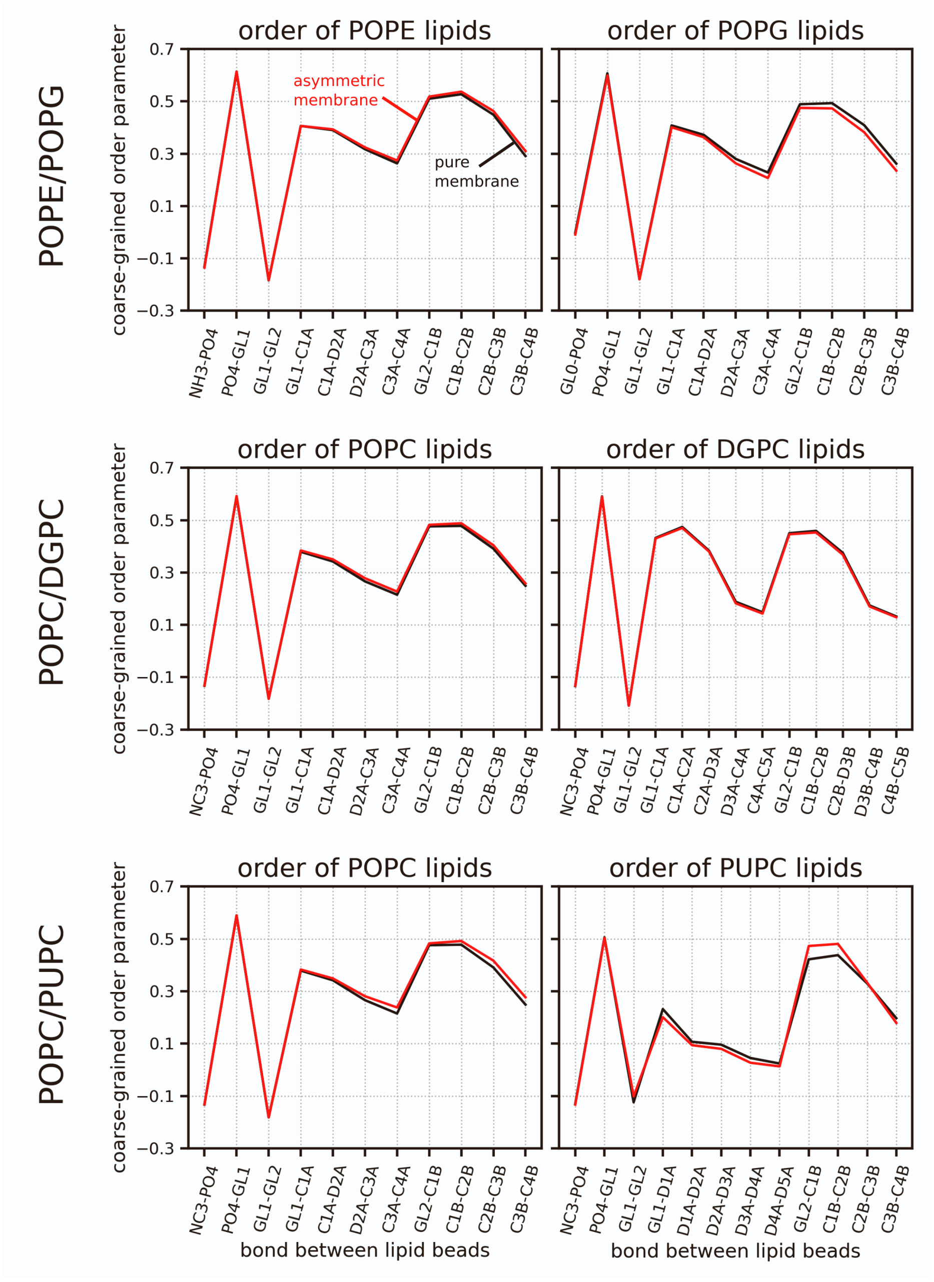

Figure S12: Coarse-grained order parameters calculated in symmetric and asymmetric membranes without the presence of a peptide. First row corresponds to membranes composed of POPE and POPG lipids. Second row corresponds to membranes composed of POPC and DGPC lipids. Third row corresponds to membranes composed of POPC and PUPC lipids. Black lines show order parameters calculated in one-component symmetric membranes composed of the concerned lipid type. Red lines show order parameters of the concerned lipid type calculated in an asymmetric membrane. The magnitude of the difference between the black and red line reflects how much the studied lipids are affected by the presence of the other leaflet, which is composed of a different lipid type, in the asymmetric membrane. Order parameters were calculated using the `order` tool available from doi.org/10.5281/zenodo.8369479.

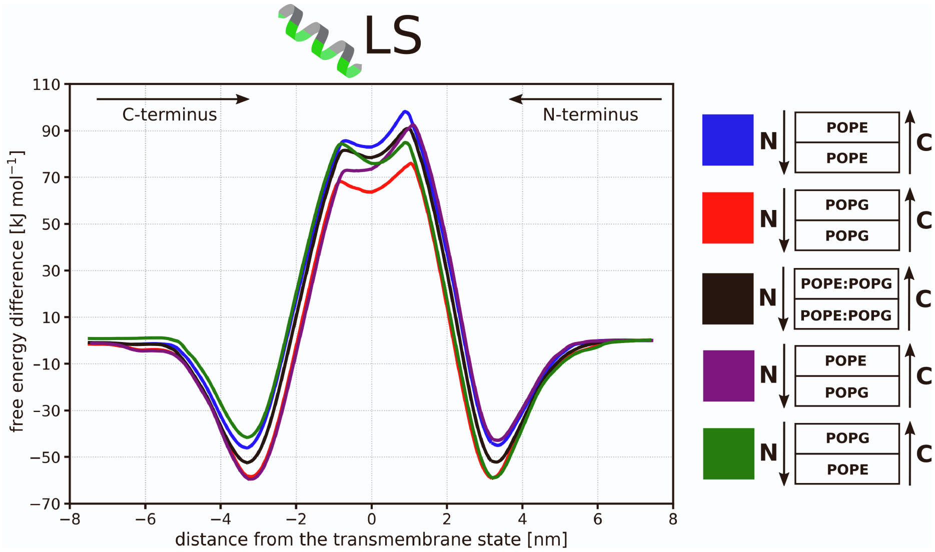

Figure S13: Free energy profiles of the LS peptide translocating through symmetric and asymmetric POPE/POPG membranes (blue = pure POPE, red = pure POPG, black = symmetric POPE:POPG 1:1, purple = asymmetric POPE/POPG, green = asymmetric POPG/POPE). The calculation error is below 5 kJ mol^{-1}. See Figure S16 for free energy profiles of the individual translocation subprocesses and convergence of the calculations.

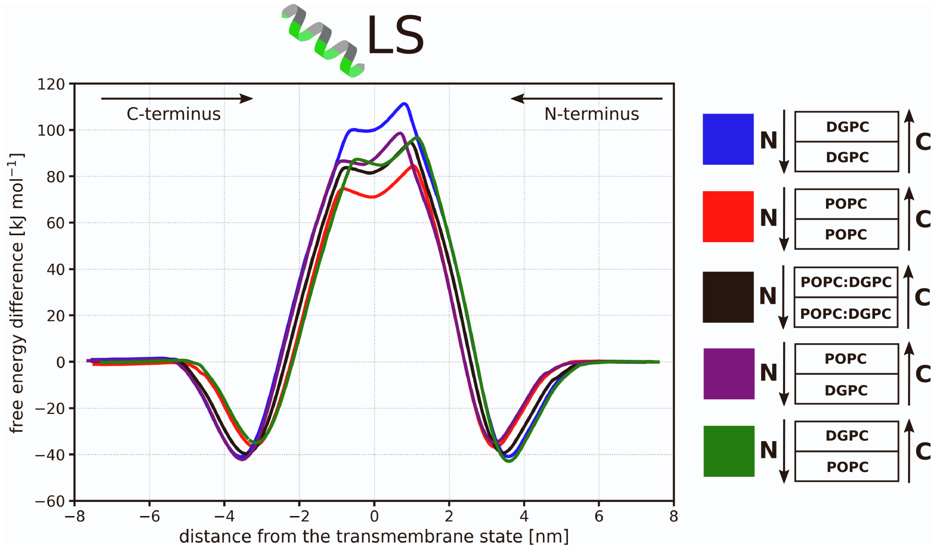

Figure S14: Free energy profiles of the LS peptide translocating through symmetric and asymmetric POPC/DGPC membranes (blue = pure DGPC, red = pure POPC, black = symmetric POPC:DGPC 1:1, purple = asymmetric POPC/DGPC, green = asymmetric DGPC/POPC). The calculation error is below 5 kJ mol^{-1}. See Figure S17 for free energy profiles of the individual translocation subprocesses and convergence of the calculations.

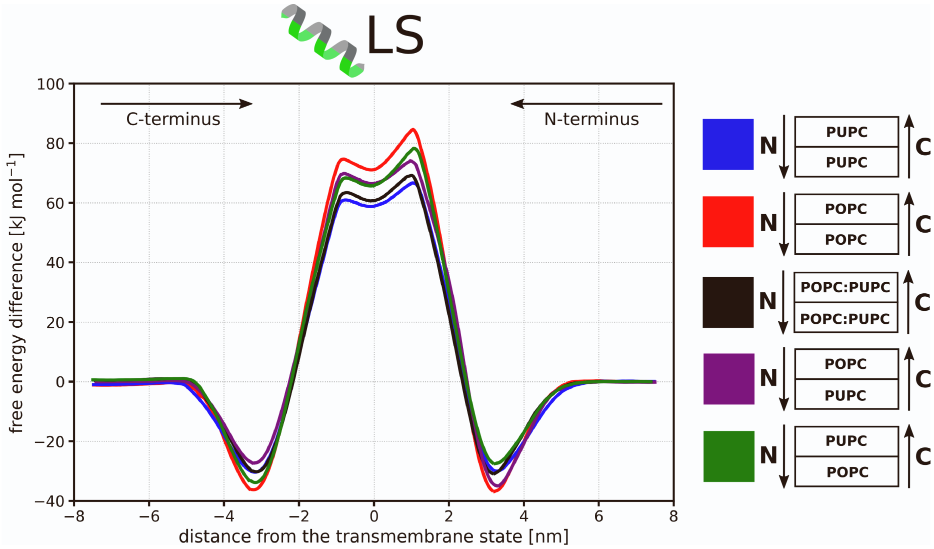

Figure S15: Free energy profiles of the LS peptide translocating through symmetric and asymmetric POPC/PUPC membranes (blue = pure PUPC, red = pure POPC, black = symmetric POPC:PUPC 1:1, purple = asymmetric POPC/PUPC, green = asymmetric PUPC/POPC). The calculation error is below 5 kJ mol^{-1}. See Figure S18 for free energy profiles of the individual translocation subprocesses and convergence of the calculations.

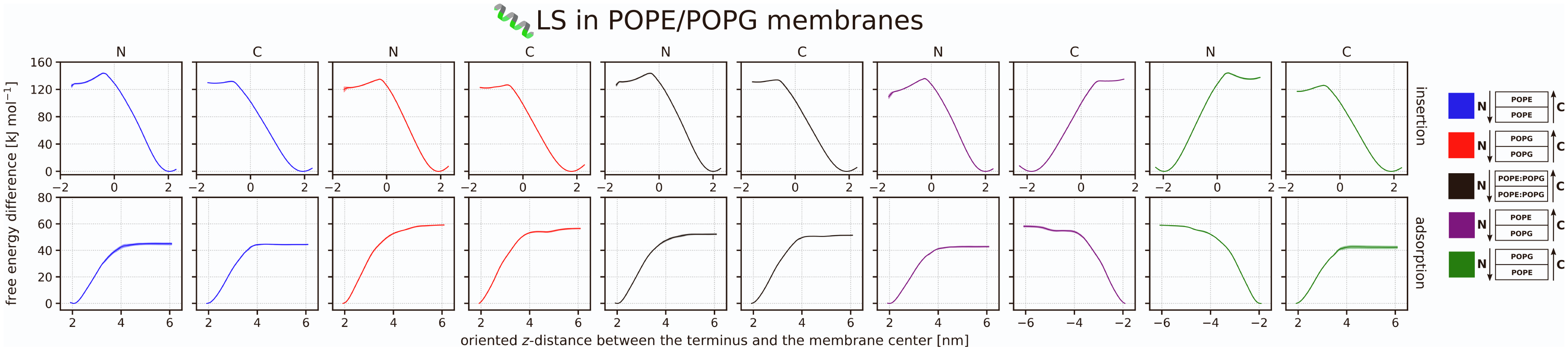

Figure S16: Free energy profiles for individual subprocesses of the LS peptide translocating through both symmetric and asymmetric POPE/POPG membranes. The full translocation profile is constructed by aligning and joining the individual subprocesses calculated for the same system, sharing the color. The color scheme is consistent with that in Figure S13. The first row presents the free energy profiles of peptide insertion, whereas the second row shows the free energy profiles of peptide adsorption. The columns labeled “N” and “C” correspond to the insertion/adsorption of the peptide’s N-terminus and C-terminus, respectively. Shading depicts the convergence of the calculation as it corresponds to the difference between the free energy profiles calculated from the initial half and the latter half of the production phase of the umbrella sampling simulation.

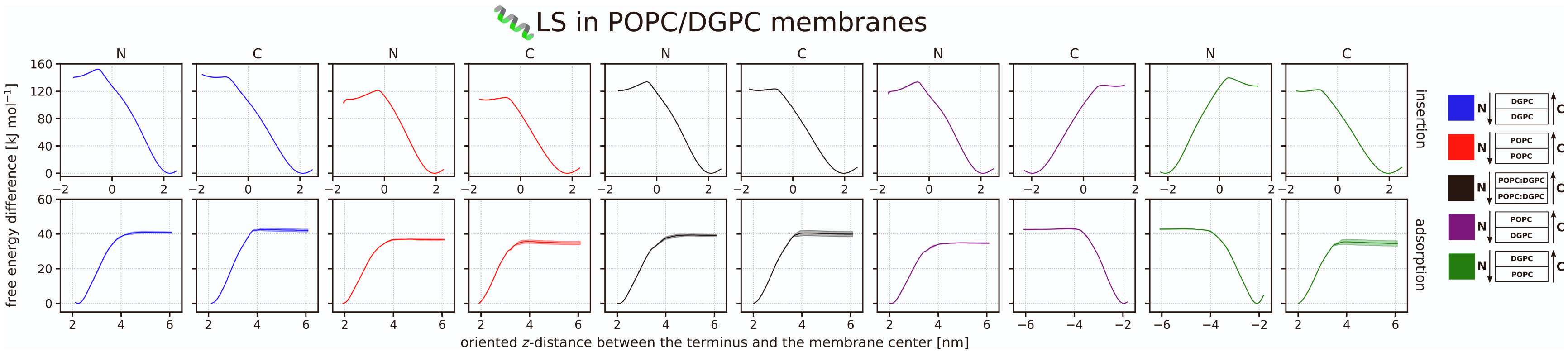

Figure S17: Free energy profiles for individual subprocesses of the LS peptide translocating through both symmetric and asymmetric POPC/DGPC membranes. The full translocation profile is constructed by aligning and joining the individual subprocesses calculated for the same system, sharing the color. The color scheme is consistent with that in Figure S14. The first row presents the free energy profiles of peptide insertion, whereas the second row shows the free energy profiles of peptide adsorption. The columns labeled “N” and “C” correspond to the insertion/adsorption of the peptide’s N-terminus and C-terminus, respectively. Shading depicts the convergence of the calculation as it corresponds to the difference between the free energy profiles calculated from the initial half and the latter half of the production phase of the umbrella sampling simulation.

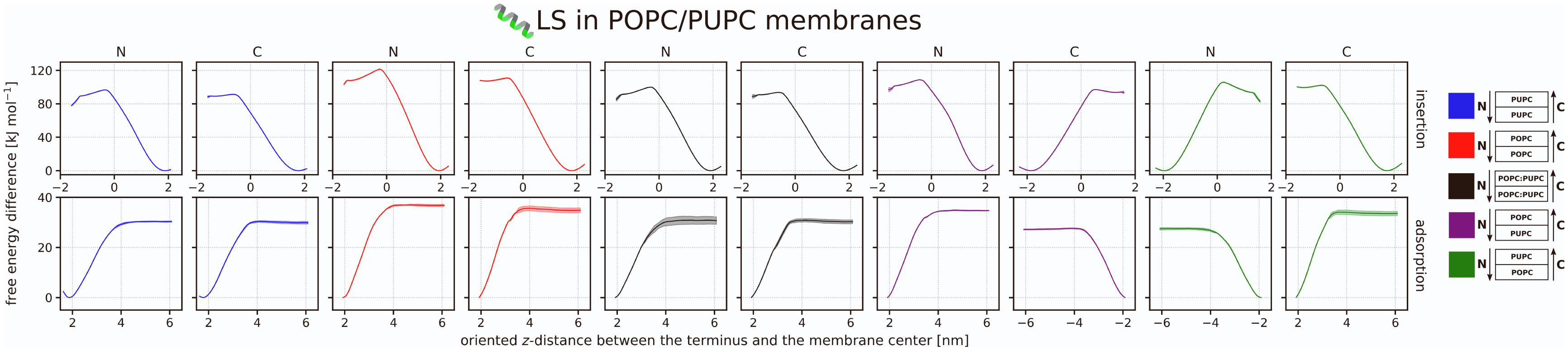

Figure S18: Free energy profiles for individual subprocesses of the LS peptide translocating through both symmetric and asymmetric POPC/PUPC membranes. The full translocation profile is constructed by aligning and joining the individual subprocesses calculated for the same system, sharing the color. The color scheme is consistent with that in Figure S15. The first row presents the free energy profiles of peptide insertion, whereas the second row shows the free energy profiles of peptide adsorption. The columns labeled “N” and “C” correspond to the insertion/adsorption of the peptide’s N-terminus and C-terminus, respectively. Shading depicts the convergence of the calculation as it corresponds to the difference between the free energy profiles calculated from the initial half and the latter half of the production phase of the umbrella sampling simulation.

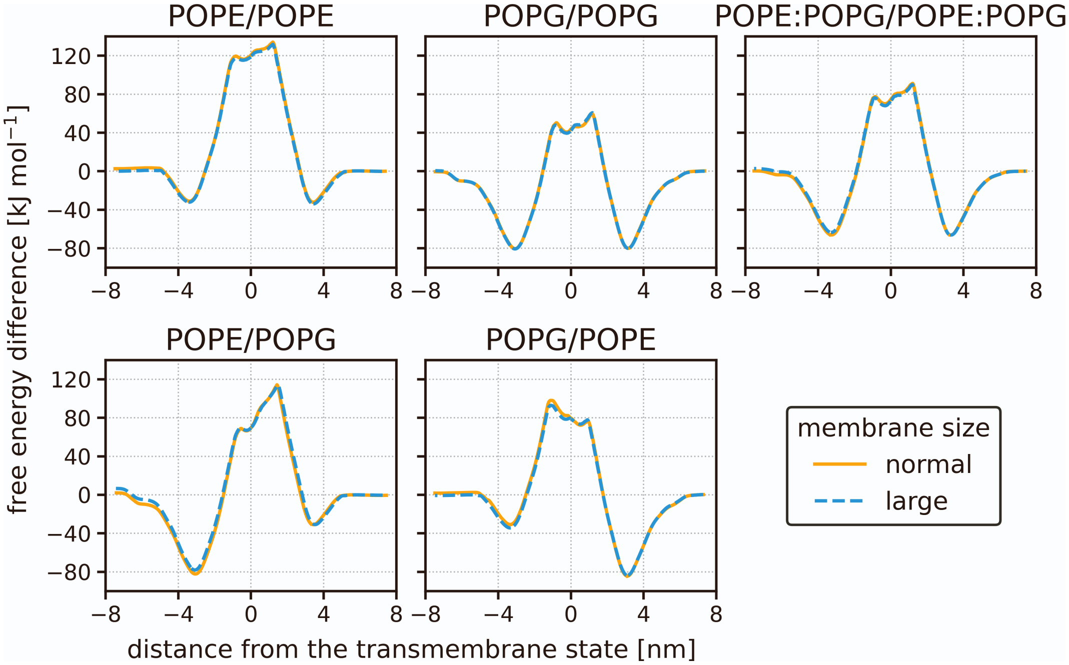

Figure S19: Free energy profiles of the LK peptide translocating through symmetric and asymmetric POPE/POPG membranes. The profiles include those for membranes composed of approximately 290 lipid molecules (normal membrane size, orange) and for larger membranes composed of roughly 970 lipid molecules (large membrane size, blue). No systematic differences were observed between the profiles calculated in normal-sized and larger membranes. This suggests that the behavior described in the main text is not an artifact of the periodic system size.

Table S5: Free energy differences for key stages in the translocation profiles of the peptides LK and LS across various symmetric and asymmetric membranes. The displayed free energy values are given relative to the peptide in solution [kJ mol^{-1}]. The error was estimated to be below 5 kJ mol^{-1}. ΔG_{AN} and ΔG_{AC} represent the free energies of the peptide's adsorbed states on either side of the membrane. ΔG_{IN} and ΔG_{IC} denote the maximum free energies when the peptide inserts its N- or C-terminus into the membrane. ΔG_{TM} corresponds to the peptide's free energy in its transmembrane state. $\Delta\Delta G_{BN}$ and $\Delta\Delta G_{BC}$ represent the translocation barriers when the peptide inserts using its N-terminus (BN) or C-terminus (BC) first. These barriers are computed by subtracting the adsorbed state's free energy (ΔG_{AN} for BN and ΔG_{AC} for BC) from the profile's maximum free energy. A higher translocation barrier signifies a more difficult translocation. See Figure S20 for graphical explanation of the free energy values of interest.

peptide	membrane	ΔG_{AN}	ΔG_{AC}	ΔG_{IN}	ΔG_{IC}	ΔG_{TM}	$\Delta\Delta G_{BN}$	$\Delta\Delta G_{BC}$
LK	POPE	−32	−31	134	120	120	166	165
	POPG	−80	−80	60	50	44	140	140
	sym POPE:POPG	−66	−66	91	77	74	157	157
	asym POPE/POPG	−31	−82	114	69	69	145	196
	asym POPG/POPE	−84	−31	80	98	80	182	129
LS	POPE	−45	−46	98	86	83	143	144
	POPG	−59	−58	76	68	74	135	134
	sym POPE:POPG	−52	−52	91	82	78	143	143
	asym POPE/POPG	−43	−59	93	74	74	136	152
	asym POPG/POPE	−59	−42	85	84	76	144	127
LS	DGPC	−41	−41	111	100	100	152	152
	POPC	−37	−36	85	75	71	122	121
	sym POPC:DGPC	−39	−40	95	84	82	134	135
	asym POPC/DGPC	−35	−42	99	87	88	134	141
	asym DGPC/POPC	−43	−35	97	87	85	140	132
LS	PUPC	−30	−30	67	61	59	97	97
	POPC	−37	−36	85	75	71	122	121
	sym POPC:PUPC	−31	−30	69	63	61	100	99
	asym POPC/PUPC	−35	−27	74	70	66	109	101
	asym PUPC/POPC	−28	−34	78	68	66	106	112

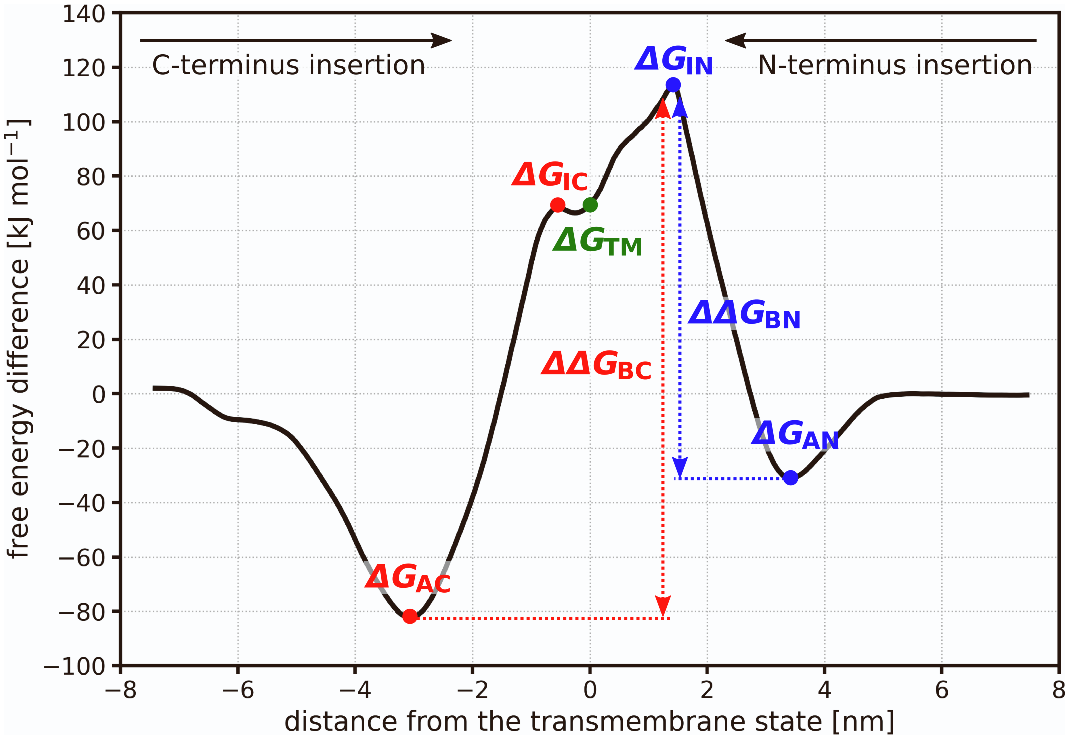

Figure S20: Free energy profile of the LK peptide translocating through asymmetric POPE/POPG membrane with important points in the profile highlighted. ΔG_{AN} and ΔG_{AC} represent the free energies of the adsorbed states of the peptide on either side of the membrane. ΔG_{IN} and ΔG_{IC} denote the free energy maxima when the peptide inserts its N- or C-terminus into the membrane. ΔG_{TM} represents the free energy of the peptide in its transmembrane state. $\Delta\Delta G_{\mathrm{BN}}$ and $\Delta\Delta G_{\mathrm{BC}}$ correspond to the translocation barriers when the peptide inserts with its N-terminus (BN) or C-terminus (BC) first. The translocation barrier is determined by the difference between the profile's free energy maximum and the free energy of the relevant adsorbed state. In this case, $\Delta\Delta G_{\mathrm{BN}} = \Delta G_{\mathrm{IN}} - \Delta G_{\mathrm{AN}}$ and $\Delta\Delta G_{\mathrm{BC}} = \Delta G_{\mathrm{IN}} - \Delta G_{\mathrm{AC}}$.

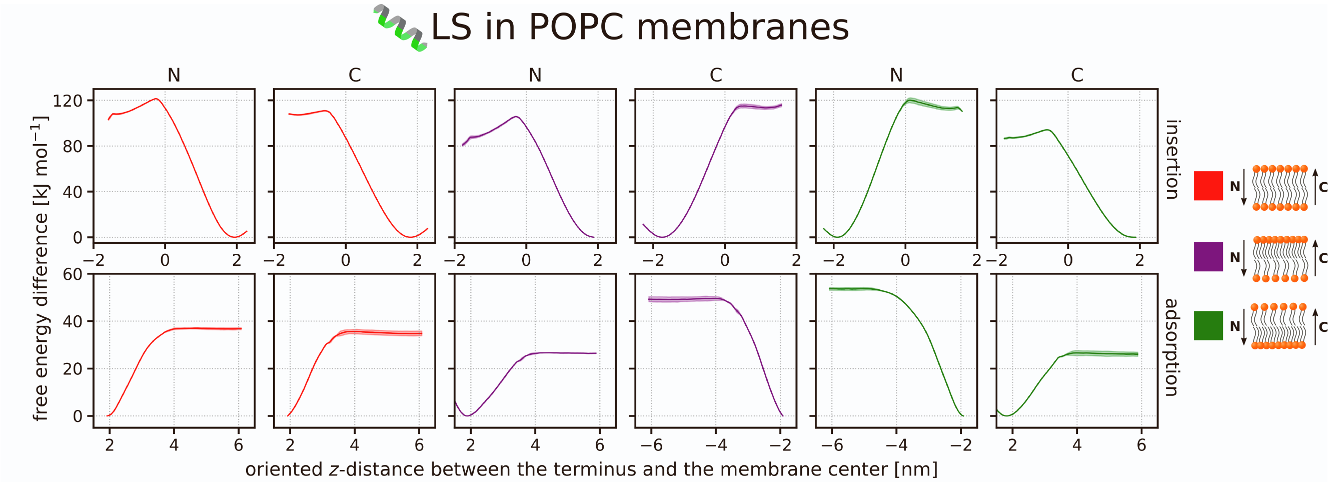

Figure S21: Free energy profiles for individual subprocesses of the LS peptide translocating through both symmetric and asymmetric POPC membranes. The full translocation profile is constructed by aligning and joining the individual subprocesses calculated for the same system, sharing the color. The color scheme is consistent with that in Figure 3. The first row presents the free energy profiles of peptide insertion, whereas the second row shows the free energy profiles of peptide adsorption. The columns labeled "N" and "C" correspond to the insertion/adsorption of the peptide's N-terminus and C-terminus, respectively. Shading depicts the convergence of the calculation as it corresponds to the difference between the free energy profiles calculated from the initial half and the latter half of the production phase of the umbrella sampling simulation.

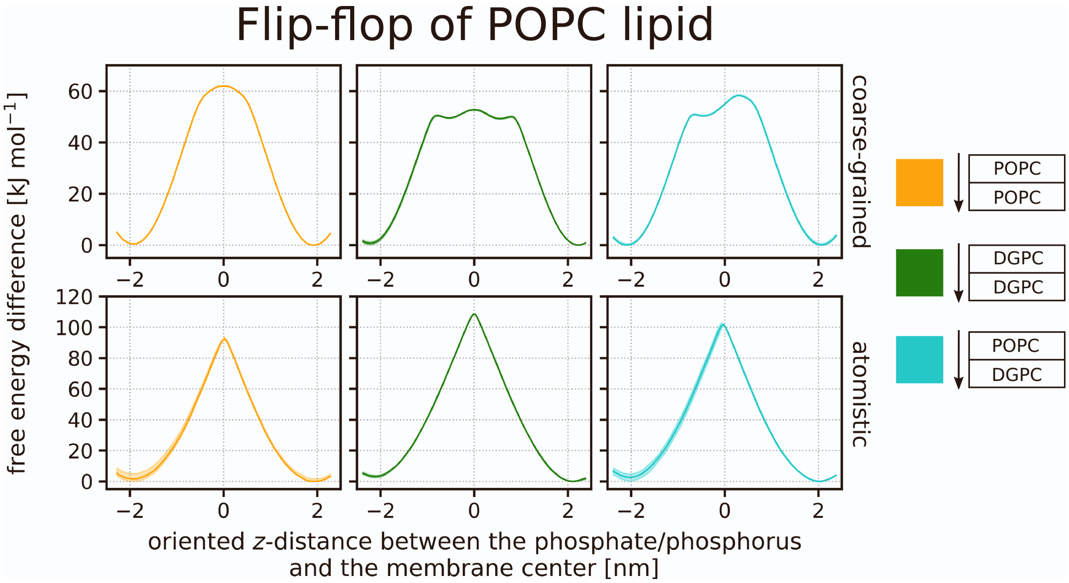

Figure S22: Free energy profiles of POPC lipid translocating through symmetric and asymmetric POPC/DGPC membranes. The color scheme is consistent with that in Figure 4. Shading is used to illustrate the convergence of the calculation. The width of the shaded area corresponds to the difference between the free energy profiles calculated from the initial half and the latter half of the production phase of the umbrella sampling simulation. A more expansive shaded region denotes a greater margin of error.
